# Combined protein and nucleic acid imaging reveals virus-dependent B cell and macrophage immunosuppression of tissue microenvironments

**DOI:** 10.1016/j.immuni.2022.03.020

**Published:** 2022-06-14

**Authors:** Sizun Jiang, Chi Ngai Chan, Xavier Rovira-Clavé, Han Chen, Yunhao Bai, Bokai Zhu, Erin McCaffrey, Noah F. Greenwald, Candace Liu, Graham L. Barlow, Jason L. Weirather, John Paul Oliveria, Tsuguhisa Nakayama, Ivan T. Lee, Matthias S. Matter, Anne E. Carlisle, Darci Philips, Gustavo Vazquez, Nilanjan Mukherjee, Kathleen Busman-Sahay, Michael Nekorchuk, Margaret Terry, Skyler Younger, Marc Bosse, Janos Demeter, Scott J. Rodig, Alexandar Tzankov, Yury Goltsev, David Robert McIlwain, Michael Angelo, Jacob D. Estes, Garry P. Nolan

**Affiliations:** 1 Department of Pathology, Stanford University, Stanford, CA, USA; 2 Vaccine and Gene Therapy Institute, Oregon Health & Science University, Beaverton, OR, USA; 3 Department of Medicine, McMaster University, Hamilton, ON, Canada; 4 Department of Otorhinolaryngology, Jikei University School of Medicine, Tokyo, Japan; 5 Division of Allergy, Immunology, and Rheumatology, Department of Pediatrics, Stanford University School of Medicine, Stanford, CA, USA; 6 Pathology, Institute of Medical Genetics and Pathology, University Hospital Basel, University of Basel, Basel, Switzerland; 7 Center of Immuno-Oncology, Dana-Faber Cancer Institute, Boston, MA, USA; 8 Department of Pathology, Brigham & Women’s Hospital, Boston, MA, USA; 9 Division of Pathobiology & Immunology, Oregon National Primate Research Center, Oregon Health & Science University, Beaverton, OR, USA; 10 Center for Virology and Vaccine Research, Beth Israel Deaconess Medical Center, Harvard Medical School, Boston, MA, USA

**Keywords:** multiplexed imaging, spatial multiomics, HIV, SIV, virus-host interactions, single-cell biology, viral pathogenesis, viral reservoir, immunosuppression

## Abstract

Understanding the mechanisms of HIV tissue persistence necessitates the ability to visualize tissue microenvironments where infected cells reside; however, technological barriers limit our ability to dissect the cellular components of these HIV reservoirs. Here, we developed protein and nucleic acid *in situ* imaging (PANINI) to simultaneously quantify DNA, RNA, and protein levels within these tissue compartments. By coupling PANINI with multiplexed ion beam imaging (MIBI), we measured over 30 parameters simultaneously across archival lymphoid tissues from healthy or simian immunodeficiency virus (SIV)-infected nonhuman primates. PANINI enabled the spatial dissection of cellular phenotypes, functional markers, and viral events resulting from infection. SIV infection induced IL-10 expression in lymphoid B cells, which correlated with local macrophage M2 polarization. This highlights a potential viral mechanism for conditioning an immunosuppressive tissue environment for virion production. The spatial multimodal framework here can be extended to decipher tissue responses in other infectious diseases and tumor biology.

## Highlights

• Development of a method (PANINI) for multiplexed nucleic acid and protein imaging
• Identification of orchestrated immune events in response to SIV infection
• IL-10 levels in B cells and macrophages correlate with immunosuppression
• Tissue microenvironmental cues can influence SIV viral transcription state

## Graphical abstract

**Figure:**
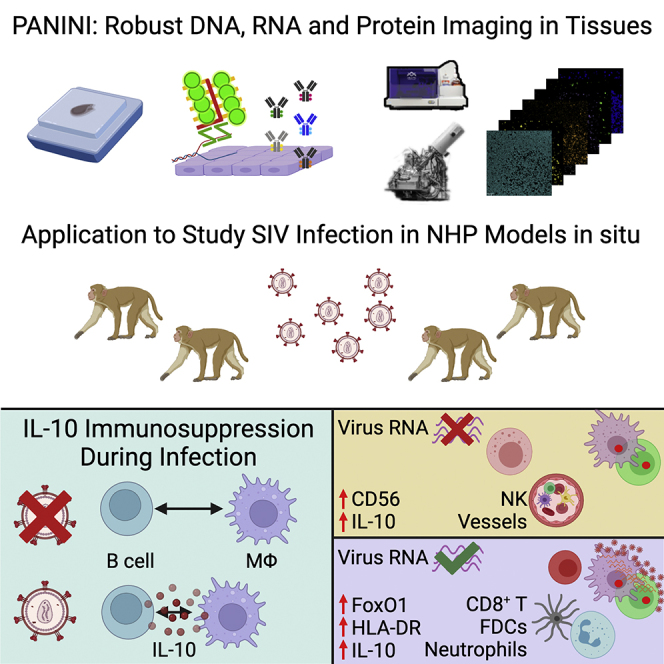

## Teaser

Our understanding of persistent HIV tissue reservoirs is constrained by our limited ability to visualize the cellular components of these viral reservoirs. Jiang et al. develop a robust method that combines nucleic acid and protein imaging, termed PANINI, and use it to uncover the orchestrated immune events of retroviral lymphoid tissue reservoirs.

## Introduction

Understanding the mechanisms and pathology of HIV persistence necessitates the ability to visualize tissue microenvironments wherein the virus resides. Current approaches to study viral tissue reservoirs often require cells to be taken out of their native tissue context (Baxter et al., 2017; Kazer et al., 2020). Complementary methods, including immunohistochemistry and *in situ* hybridization (ISH) technologies, are compatible with formalin-fixed paraffin-embedded (FFPE) pathogen-inactivated tissues to retain information in 2D but are constrained by the low number of concurrently detectable features (Deleage et al., 2016; Estes et al., 2017). Multiplexing markers on a single tissue section is routine using immunofluorescence (IF) microscopy but is limited by factors such as the spectral overlap of fluorophores and incompatible host species of primary antibodies. Recent advances in multiplexed imaging modalities, such as multiplexed ion beam imaging (MIBI) (Angelo et al., 2014; Keren et al., 2018), co-detection by indexing (CODEX) (Goltsev et al., 2018; Schürch et al., 2020), imaging mass cytometry (IMC) (Giesen et al., 2014), signal amplification by exchange reaction (SABER) (Kishi et al., 2019; Saka et al., 2019), and cyclic immunofluorescence (cycIF) (Lin et al., 2015, 2018), either utilize iterative methods (CODEX, SABER, and cycIF) or mass spectrometry (MIBI and IMC) to overcome these challenges. Highly multiplexed *in situ* detection of mRNA and protein epitopes in archival samples has also been achieved with a branched-chain amplification method coupled with antibody-based detection (Schulz et al., 2018; Wang et al., 2012), but this procedure has been validated only for highly abundant RNA transcripts, and robust protein epitope detection is hindered by the required protease treatment step (Schulz et al., 2018).

The ability to simultaneously detect nucleic acids present at low abundance, such as a single copy of the DNA resulting from a viral integration event and protein molecules *in situ*, is paramount for enabling studies of viral infection and beyond. We reasoned that combining a customized branched-chain amplification method with the covalent deposition of haptens would enable multiplexed imaging on various antibody-based platforms, including MIBI and CODEX. By turning nucleic acid detection into an antibody “problem,” we may thus overcome the limited sensitivity of ISH in tissues. For example, on the IMC and MIBI platforms, each oligonucleotide-based probe can only carry a maximum of 20 metal ions (Frei et al., 2016), whereas each antibody has a theoretical capacity for ∼100 metal ions (Bendall et al., 2011; Han et al., 2018).

Here, we present an approach, protein and nucleic acid *in situ* imaging (PANINI), that consists of a (1) highly sensitive custom branched-chain amplification method for nucleic acid targets using a tyramide-based amplification to deposit haptens, which are then detected using antibodies conjugated to lanthanide or oligo tags for a further amplification step, (2) an optimized antigen retrieval protocol that bypasses the protease treatment and yet allows nucleic acid detection down to a single genomic event, and (3) an antibody-based detection of nucleic acid targets and proteins by using the MIBI (Keren et al., 2019), Vectra Polaris (Hoyt, 2021), and CODEX platforms (Goltsev et al., 2018). Using FFPE cell pellets and lymphoid tissues from simian immunodeficiency virus (SIV)-infected and SIV-uninfected rhesus macaques, we demonstrate that PANINI-MIBI is capable of simultaneous detection of single-integration events of SIV DNA (vDNA), SIV RNA transcripts (vRNA), and protein epitopes robustly on the same tissue section.

We utilized PANINI-MIBI to characterize the viral reservoir and immune responses within SIV-infected and SIV-uninfected control lymphoid tissues. The tissue immune responses to lentiviral infection were heterogeneous, and phenotypically similar cells from infected animals and uninfected controls exhibited substantially different functions. For instance, interleukin 10 (IL-10) expression was increased in B cells upon infection, thus promoting a postulated polarization of macrophages to an immunosuppressive M2 phenotype, which was correlated to a known conducive environment for SIV transcriptional activation. Characterization of the higher order structure around infected cells that are virus transcriptionally silent or active revealed microenvironmental differences, enabling us to propose a model for how chronic SIV infection dampens the immune response and to elucidate the coordinated host features that may affect viral transcription state in tissue reservoirs. This work provides a framework for future multimodal studies of the principles of host-pathogen interactions *in situ* using inactivated archival tissue samples and can also be broadly applied to understand the spatial context of other elements in health and disease.

## Results

### Development of a sensitive approach to combine nucleic acid and protein detection in archival tissue samples

**Figure 1:**
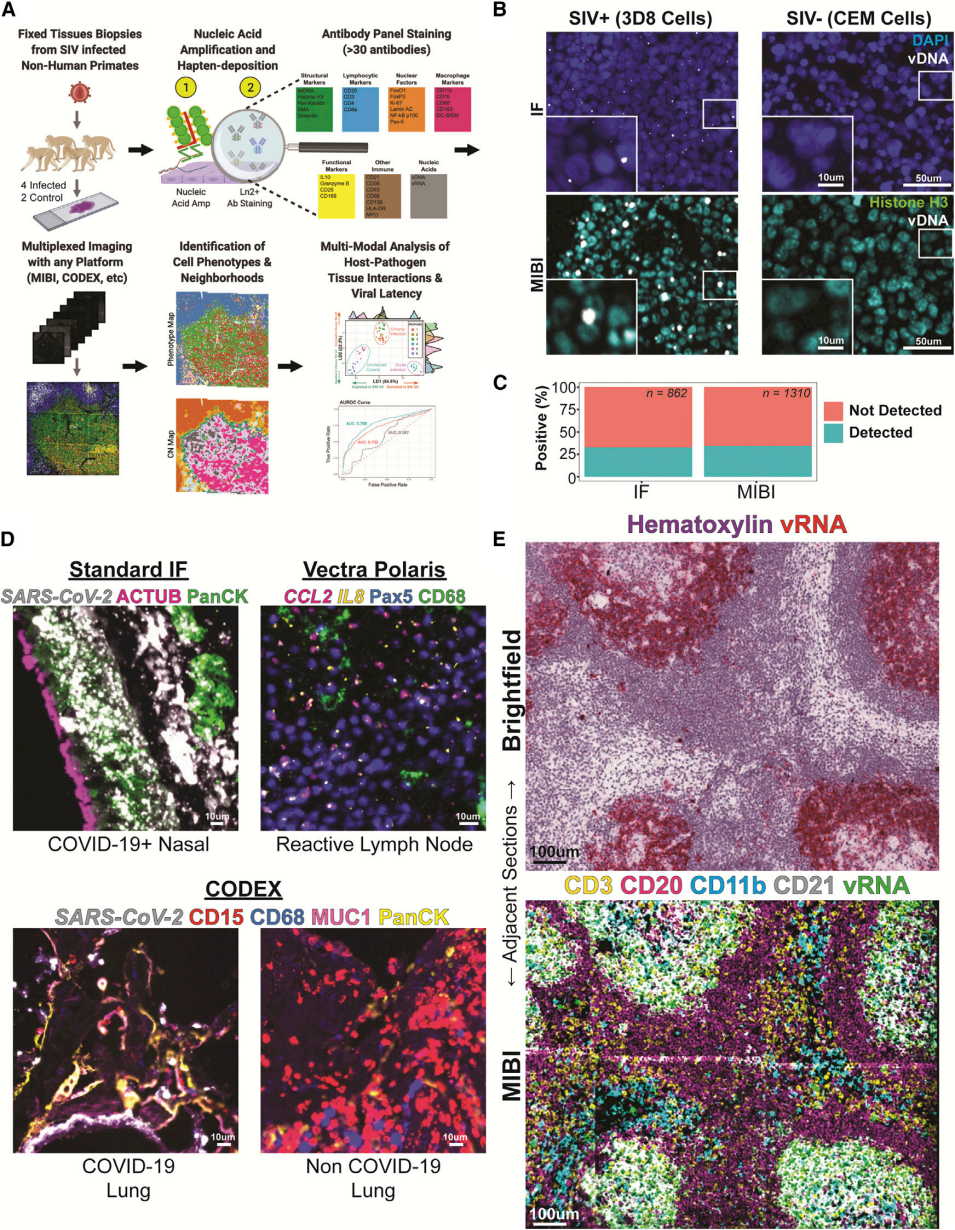
PANINI enables sensitive multiplexed, strand-specific nucleic acid and protein detection in archival tissues (A) An overview of the experimental workflow and analytical framework for PANINI. In short, tissue autopsy sections (4 SIV-infected and 2 SIV-uninfected rhesus macaques for this study) are subject to PANINI, which couples nucleic acid amplification and peroxidase-catalyzed hapten deposition with antibody-based detection of both nucleic acid and protein targets. Multiplexed images were then acquired using multiplexed imaging platforms such as MIBI and CODEX and computationally analyzed for a high-resolution understanding of host-pathogen tissue interactions *in situ*. (B) Representative IF and MIBI images of positive control 3D8 and negative control CEMs cell pellet sections stained in the same experiment. Nuclear stains, DAPI for IF and Histone H3 for MIBI, are in blue and cyan, respectively; vDNA is in white. (C) Quantification of 3D8 cells from (B) that are positive for vDNA signals. (D) Cross-platform compatibility of PANINI. Top Left: standard immunofluorescence on COVID-19 positive nasal tissues for the SARS-CoV-2 spike mRNA (*SARS-CoV-2*, white), cilia marker acetylated tubulin (ACTUB, magenta), and epithelia marker pan-cytokeratin (PanCK, green). Top Right: Vectra Polaris on reactive lymph nodes and cytokine transcripts (*CCL2*, magenta; *IL8*, yellow), the B cell marker (Pax5, blue), and the macrophage marker (CD68, green). Bottom: CODEX on COVID-19 positive (left) and negative (right) lung samples for the SARS-CoV-2 spike mRNA (SARS-CoV-2, white), neutrophil marker (CD15, red), macrophage marker (CD68, blue), type II pneumocytic marker (MUC1, magenta), and epithelia marker pan-cytokeratin (PanCK, yellow). (E) Adjacent sections from an SIV-positive lymph node. Both slides underwent PANINI treatment. For the top section, a fast red chromogenic substrate was used for vRNA (red) with hematoxylin. The bottom section was stained with a MIBI-compatible antibody cocktail. Markers shown here delineate specific cell lineages: CD3 (T cells, yellow), CD20 (B cells, purple), CD11b (monocytes, blue), CD21 (B cells and FDCs, white), and SIV vRNA (green). See also Figure S1.

PANINI was designed to be analogous to routine ISH and immunohistochemistry, with the addition of adapting nucleic acid imaging for antibody-hapten detection for downstream compatibility with high-dimensional tissue imaging platforms and multimodal analysis (Figure 1A). We first tested this approach using both IF and MIBI on 3D8 cells containing a single integration of SIV vDNA, and the SIV-negative CEM cells from which they were derived (Nishimura et al., 2009). We observed positive vDNA foci only in the 3D8 cells (Figure 1B). Quantification of vDNA-positive puncta across IF and MIBI data (∼28%) aligned with previous studies, in which there is a 21%–29% probability of capturing a positive nuclear event due to the thickness of the cell pellet sections (4–6 μm) being thinner than the average size of the 3D8 and CEM cells (15 μm) (Deleage et al., 2016). This high concordance is indicative of the applicability of PANINI for sensitive, targeted detection of nucleic acids, down to a single SIV integration event, in FFPE archival tissue samples.

The protease digestion step is used in various ISH assays (Deleage et al., 2016; Wang et al., 2012) to increase the accessibility of target nucleic acids by disrupting the packed architecture of tissue matrixes and nucleic acid-binding proteins (Yang et al., 1999). We found that a pH 9 antigen retrieval step allowed detection of vDNA and vRNA in FFPE lymph node sections from an SIV-infected rhesus macaque without the need for protease digestion using via IF and MIBI (Figure S1A). These results show that PANINI retains ISH sensitivity, down to a single copy, although preserving protein epitopes for downstream antibody assays.

### Flexible and robust compatibility of PANINI for multiplexed imaging platforms

PANINI was additionally compatible with standard IF, multispectral imaging with the Vectra Polaris, and the antibody-oligo based CODEX platforms. We combined PANINI for SARS-CoV-2 spike mRNA detection with acetylated tubulin (ACTUB; cilia marker) and pan-cytokeratin (PanCK; epithelium marker) on a standard IF microscope on COVID-19 nasal autopsies. We observed the rampant presence of SARS-CoV-2 spike transcripts throughout the nasal epithelium near ciliated cells, the postulated primary nasal route of entry for the virus (Figure 1D; Lee et al., 2020). Of note is the preserved nasal epithelium cilia staining, a delicate organelle easily disrupted by proteases.

We also detected the cytokine transcripts *CCL2* and *IL8*, with protein targets CD68 (macrophages) and Pax5 (B cells) within human reactive lymph nodes using multispectral whole-slide scanning (Figure 1D). PANINI is also compatible with CODEX multiplexed imaging, exemplified using COVID-19 lung samples or negative controls (Figures 1D and S1B). Here, we combined SARS-CoV-2 spike mRNA detection with protein targets CD15 (neutrophils), CD68 (macrophages), MUC1 (Type II pneumocytes), and PanCK (epithelia cells). The PANINI-CODEX approach was validated against standard brightfield single-plex ISH on tissue samples containing high, low, and no SARS-CoV-2 (Figure S1B). These results establish PANINI as a versatile experimental framework for sensitive detection of nucleic acid and protein targets in archival tissue samples in a platform-agnostic fashion.

### Development of a PANINI-MIBI nonhuman primate panel to study SIV infection

To better analyze the dynamic immune response to SIV infection, we validated and applied a 33-marker panel, including probes to SIV DNA and RNA (Figure S1C), across lymphoid tissues from four SIV-infected and two uninfected rhesus macaques, resulting in 464,248 spatially resolved cells (Table S1). This panel was purposefully designed to encompass tissue and cellular structures (dsDNA, Histone H3, Pan-Keratin, SMA, and Vimentin), functional nuclear (FoxO1, FoxP3, Ki-67, Lamin AC, NF-κB-p100, and Pax-5) and cytoplasmic markers (IL-10, granzyme B, and CD25), proteins to delineate monocytic and macrophage functions (CD11b, CD16, CD68, CD163, and DC-SIGN), markers to phenotype immune cell populations (CD21, CD36, CD45, CD56, CD138, HLA-DR, and MPO), and SIV-infected cells that are transcriptionally active (vDNA^+^ and vRNA^+^) or silent (vDNA^+^ and vRNA^−^). Each of these markers were thoroughly assessed for their staining specificity, distribution, and potential off-target staining (Figure S1D) and were further cross-validated against published immunohistochemistry studies, manufacturer data sheets, and the human protein atlas database (Uhlen et al., 2017). Conjugated antibodies were then carefully titrated to ensure no observable signal bleed through of hydrides (+1), oxides (+16), and hydroxides (+17) of the lanthanide antibody tags (Keren et al., 2019). Adjacent sections of a lymph node from an SIV-infected rhesus macaque were subject to standard single-plex brightfield RNAscope ISH (Figure 1E, top) or PANINI-MIBI (Figure 1E, bottom; Figure S1E), demonstrating that the latter captures equivalent viral events (SIV vRNA) and tissue morphology while substantially expanding upon the measured markers. Representative lineage-specific markers delineate some of the diverse immune cells present: CD3 (T cells), CD20 (B cells), CD11b (monocytes), and CD21 (B cells and follicular dendritic cells [FDCs]) (Figure 1E, bottom). Multiplexed antibody imaging platforms thus present the ability to (1) cross-validate antibody markers present or absent on specific cell lineages against each other to attain high levels of stringency (Keren et al., 2018; Phillips et al., 2021) and (2) harness both phenotyping and functional markers to understand both cell identity and function (Figure S1D). These include regulatory T cells (Tregs; Figure S1F), granzyme B^+^ CD8^+^ T cells (Figure S1G), B cells and FDCs (Figure S1H), and M1/M2 macrophages (Figure S1I).

### Scalable automated cell segmentation and cell type identification to assess orchestrated immune responses

**Figure 2:**
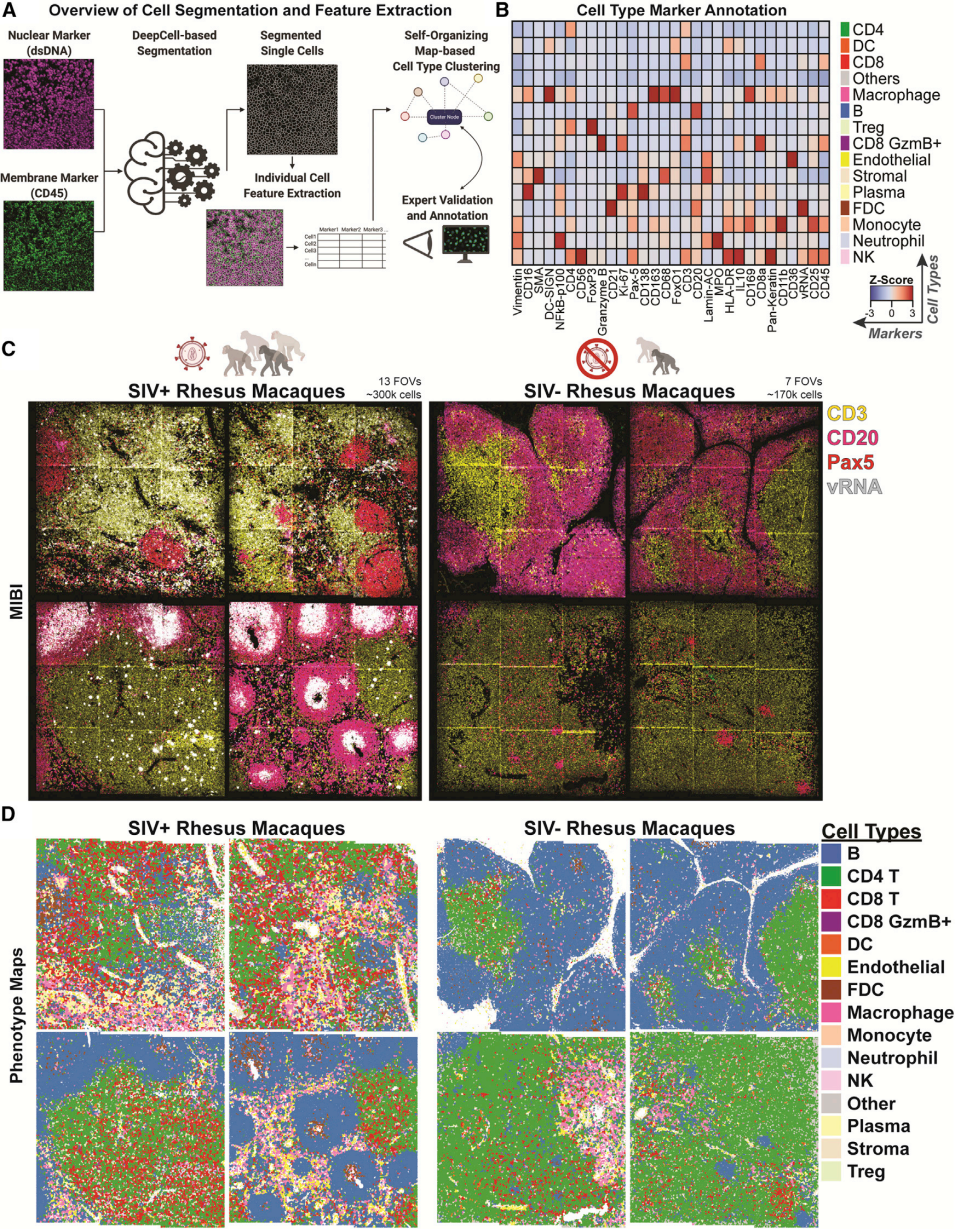
Unsupervised computation methods for rapid feature extraction and phenotypic identification from multiplexed imaging data (A) An overview of the deep learning-enabled segmentation, feature extraction, and self-organizing map-based cell type clustering and annotation used in this study. (B) A heatmap depicting the *Z* scores of marker expression and cell types identified in all FOVs. (C) Representative FOVs of tissues from SIV-infected and control animals pseudocolored to show regions enriched in B and T cells and in SIV vRNA. Each FOV is 1.2 × 1.2 mm, with 20 FOVs acquired across four SIV-infected and two SIV-uninfected rhesus macaques to generate ∼470,000 spatially resolved cells. (D) Individual cells from the representative FOVs in (C) colored by their cellular phenotypes. See also Figure S2.

Accurate cell segmentation methods are required to confidently extract single-cell feature information from multiplexed tissue images (Hollandi et al., 2020; Moen et al., 2019; Van Valen et al., 2016). We used Mesmer and FlowSOM for cell type segmentation and clustering, respectively (Figure 2A; Gassen et al., 2015; Greenwald et al., 2021). We identified 14 distinct immune and structural cell types, with the expected associated lineage-specific marker expression (Figure 2B). Visual inspection of the MIBI-multiplexed images (Figure 2C) and their paired spatial phenotype maps confirmed accurate cell type annotation (Figure S2A). We orthogonally performed immunofluorescent immunohistochemistry on three consecutive sections juxtaposed to the original PANINI-MIBI analyzed section to confirm specificity and scalability of the unsupervised cell annotation methodology (Figure S2B). Prominent tissue features, including B cell follicles, T cell zones, and the macrophage-rich medullary sinus, were visible on both the raw MIBI images (Figure 2C) and phenotype maps (Figures 2D and S2C), further confirming the robustness of the cell segmentation and annotation methodology.

**Figure 3:**
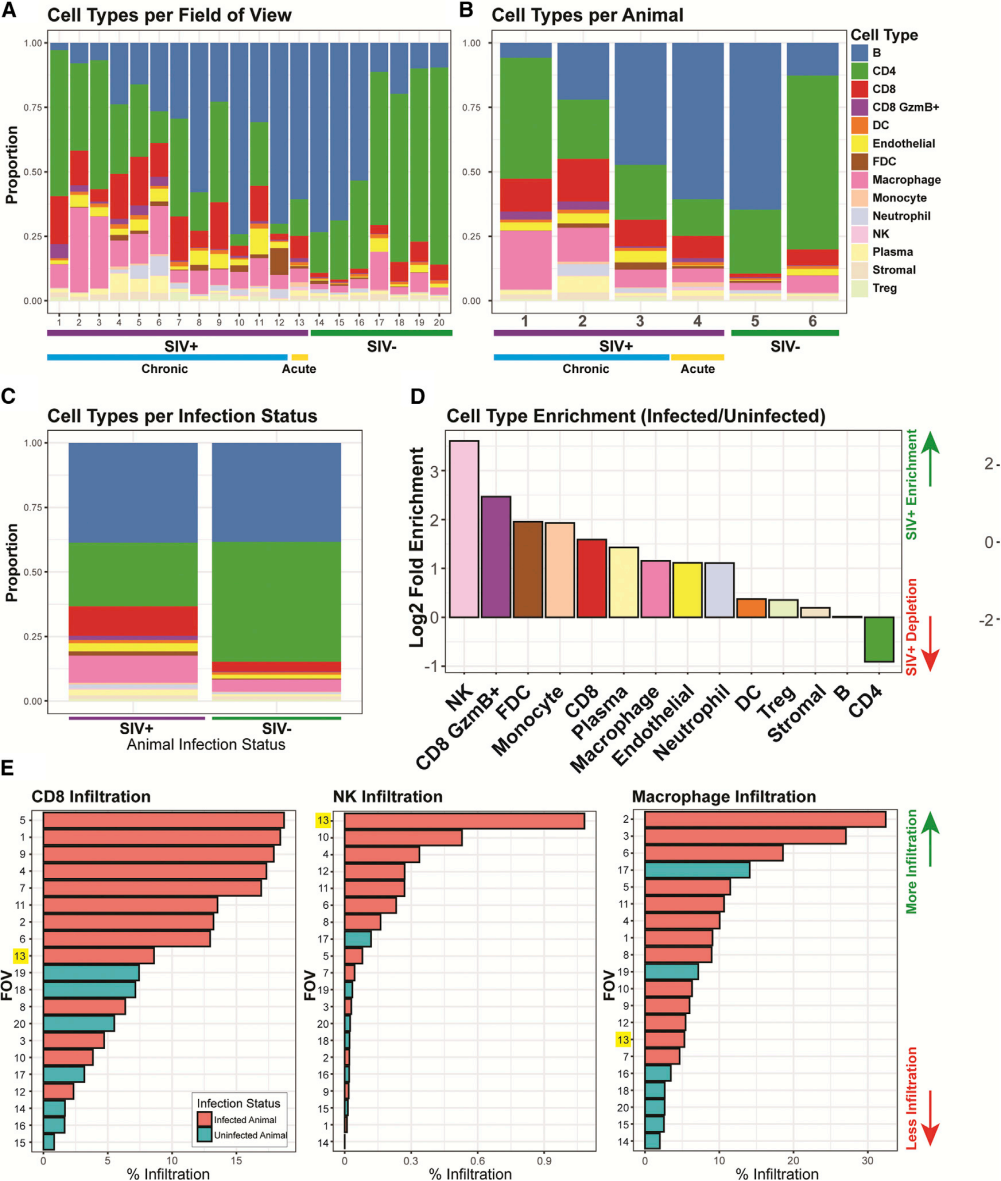
Spatial dissection of orchestrated immune composition and responses to SIV infection (A) Bar plots of proportions of each cell type per FOV across the 20 FOVs acquired in this study. (B) Bar plots of proportions of each cell type aggregated on a per animal basis. (C) Bar plots representing the proportions of each cell type aggregated by infection status. (D) Ranked log_2_-fold enrichment (infected over uninfected controls) for each cell type, ranked from the most enriched (left) to most depleted (right) in SIV-infected animals relative to uninfected controls. (E) Ranked bar plots showing the percent infiltration of each cell type indicated across the 20 FOVs with bars colored by infection status. The yellow box indicates the acutely infected animal. See also Figure S3.

We next analyzed the summary statistics of the 14 different cell types based on the individual FOVs (Figure 3A), animals (Figure 3B), and infection status (Figure 3C). There was an evident depletion of CD4^+^ T cells in SIV-infected animals (Figure 3D), a hallmark of HIV-1 and SIV infection (Estes et al., 2008; Picker, 2006; Zeng et al., 2011). B cell numbers were relatively stable, but the infiltration of other immune cell types, such as NK cells, CD8^+^ T cells, FDCs, and macrophages, was increased upon infection (Figure 3D). On the individual FOV basis, the amounts of CD8^+^ T cell, NK cell, and macrophage infiltrations were highly correlated with the infection status of the animal, indicative of the host immune response (Figure 3E). This was also observed for CD8^+^ granzyme B^+^ T cells, dendritic cells, endothelial cells, FDCs, monocytes, neutrophils, and plasma cells (Figure S3). Extensive SIV deposition was seen on FDCs within B cell follicles in FOVs from SIV-infected tissues, reflective of FDC expansion during infection (Figures 2E, S2C, and S3). These responses suggest higher-order coordination between cell types, beyond phenotypic measurements of individual cells.

### Cellular neighborhoods reflect changes in tissue microenvironments upon viral infection

**Figure 4:**
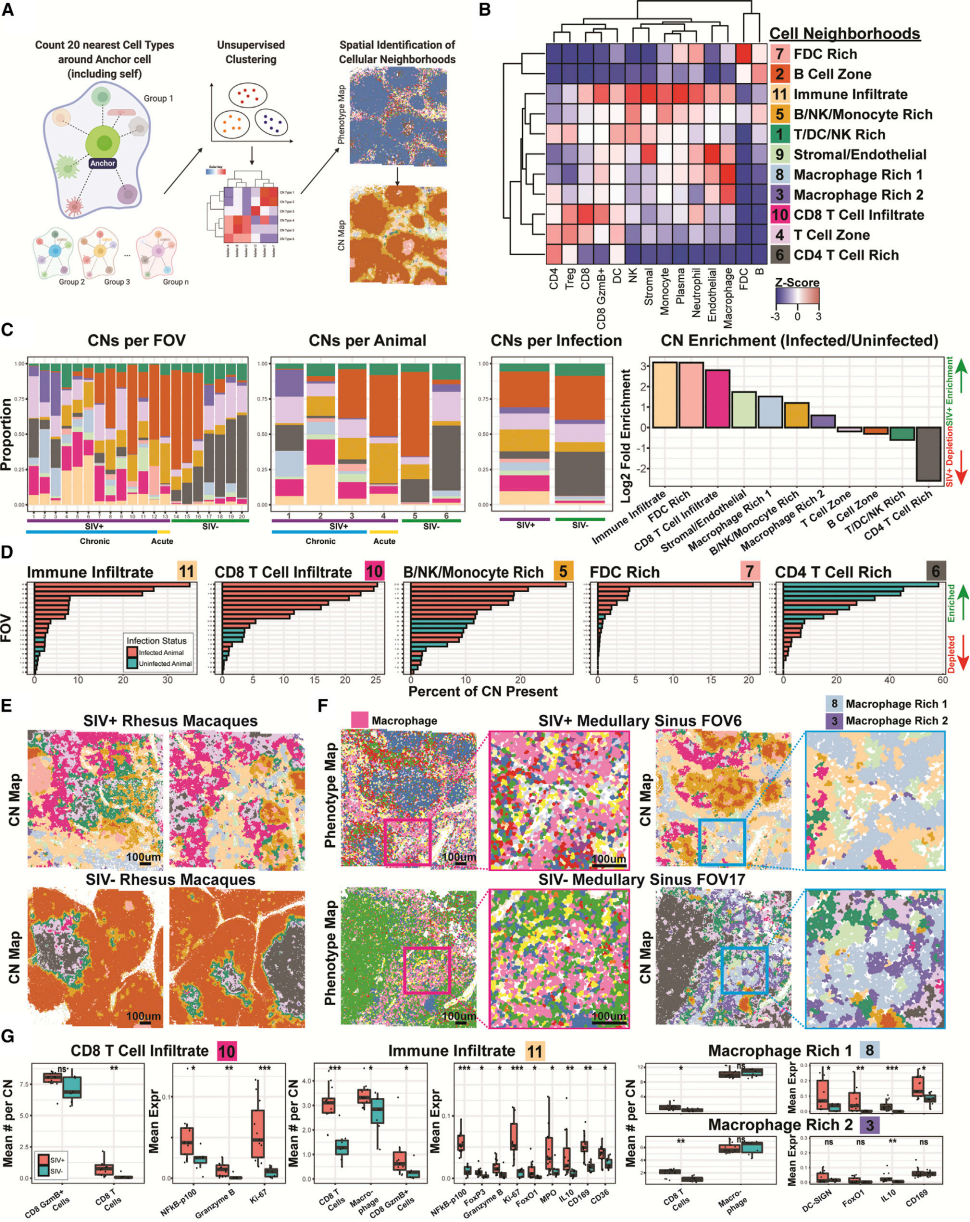
Cellular neighborhood analysis enables functional stratification of tissue microenvironments during viral infection (A) Overview of the method used to define CNs. Twenty nearest neighboring cells (including itself) around each cell were defined, and the cell types were quantified and subjected to unsupervised clustering to define CNs. (B) A *Z* score heatmap depicting the 11 CNs identified and cell type enrichment. (C) From left to right, proportions of each CN aggregated by FOV, animal, and infection status and plot of ranked log_2_-fold enrichment (infected over uninfected controls) for each CN. (D) Ranked bar plots showing the percent composition of each CN across the 20 FOVs with bars colored by infection status. (E) Representative FOVs of infected and control animals (also shown in Figures 2C and 2D) with each individual cell colored by CNs. (F) Representative FOVs from SIV-infected (top) and SIV-uninfected (bottom) animals containing medullary sinus regions depicted as phenotype maps (left) and CN maps (right). Red and blue boxes indicate regions magnified for zoomed-in views of macrophage-enriched regions. Pink cells in the phenotype map are macrophages. Light blue and purple CNs 8 and 3, respectively, are macrophage rich. (G) Box plots of mean numbers for cell types within CNs (left) and the mean expression of functional markers within the CN (right). Each dot in the box plot represents data from a single FOV, and the data are divided between infected (orange) and healthy controls (teal). Nonpaired Wilcoxon test: ns, not significant; ^∗^p < 0.05; ^∗∗^p < 0.01; ^∗∗∗^p < 0.001. See also Figure S4.

Tissue microenvironments are dynamic amalgamations of multiple cell types with ranges of functions within an organ system, governed by local tissue context such as the immune cell and pathogen composition. Unlike tissue morphologies, which are structural determinants of tissue architecture and the associated cell types (Xu et al., 2009), the tissue microenvironment can be approximated as an accumulation of various chemical and biological determinants exerted both by and onto cells in its native context. Here, we adopted the empirical cellular neighborhood (CN) methodology to quantitatively define the microenvironments of healthy and SIV-infected tissues (Schürch et al., 2020). Each cell within a tissue is sequentially “anchored,” and cellular phenotypes are tabulated for the nearest 19 cells around itself (for a total of 20 cells). These “bags” of 20 cells were then subject to unsupervised k-means clustering (see Figure 4A and material and methods for more details). CNs take into consideration the impact of the cellular identity of surrounding neighbors on the function of the anchor cell (Figure 4A). Importantly, neither the infection status nor phenotypic and functional markers (e.g., CD4, Ki-67, CD169, and FoxO1) were considered in defining CNs; therefore, the microenvironment was defined using only the spatial phenotypic patterns. We thus identified 11 distinct CNs with stratified cell compositions (Figure 4B): T cell-, dendritic cell-, and NK cell-rich CN1; B cell zone-containing CN2; macrophage-rich CN3; T cell zone-containing CN4; B cell-, NK cell, and monocyte-rich CN5; CD4^+^ T cell-rich CN6; FDC-rich CN7; macrophage-rich CN8; stromal and endothelial enriched CN9; CD8^+^ T cell infiltrate-containing CN10; and immune infiltrate-containing CN11. CN summary statistics (Figure 4C) reflect similar trends as the phenotype summary statistics (Figures 3A–3D), albeit with additional stratification of cell types such as the CD4^+^ T cells. The ranking of CNs for each FOV revealed the enrichment of certain CNs, including as CNs 5, 7, 10, and 11, in SIV-infected animals (Figures 4D and S4A) and the depletion of the CD4^+^ T cell-rich CN6 (Figures 4C and 4D).

The CN maps are reflective of tissue properties with an additional dimension of information beyond the phenotype maps (Figure 2D, top row, and S3 for phenotype maps; Figures 4E and S4B for CN maps). For example, although macrophages predominate in the phenotype maps in SIV-infected and SIV-uninfected FOVs, the CN maps for the same areas show a more complex picture with the presence of two different macrophage-rich CNs, CN3 and CN8 (Figure 4F). CN3 (enriched for macrophages and CD4^+^ T cells) is more dominant in SIV-negative FOVs, whereas CN8 (enriched for macrophages, neutrophils, and CD8^+^ granzyme B^+^ T cells) is the predominant CN in the SIV-positive FOVs. This highlights that cellular functions are influenced by surrounding external factors.

Quantification of the average SIV vRNA for each CN showed that the FDC-rich CN7 had the highest quantities of vRNA (Figure S4C), consistent with FDCs’ function of long-term retention of antigen-immune complexes (including HIV/SIV) within the B cell follicles and augmentation of humoral responses by “presenting” native antigens to B cells during the germinal center reaction (Burton et al., 2002). Individual CNs also differed between SIV-infected and SIV-uninfected conditions; there were more CD8^+^ T cells in SIV-infected tissues than healthy controls detected in the CD8^+^ T cell infiltrate-heavy CN10. We also observed increased CD8^+^ T activation markers, such as NF-κB-p100, granzyme B, and Ki-67 during infection in CN10 (Figure 4G, left). In CN11, which is characterized by immune cell infiltrates, both adaptive and innate immune cells (e.g., CD8^+^ T cells and macrophages) and functional proteins marker (e.g., NF-κB-p100, FoxP3, granzyme B, Ki-67, FoxO1, MPO, IL-10, CD169, and CD36) were elevated in SIV-infected samples compared with uninfected controls (Figure 4G, middle).

Although there were no significant differences between the mean number of macrophages due to infection in the two macrophage-associated CNs (CN8 and CN3), CD8^+^ T cell abundance was slightly higher in SIV-infected tissue samples (Figure 4G, right). We further observed the dominant expression of macrophage-functional markers (DC-SIGN, FoxO1, IL-10, and CD169) in CN8, but only slightly higher expression of IL-10 in CN3 pertaining to infected tissues. This suggests that SIV infection induces tissue-specific responses in macrophages, and higher DC-SIGN, FoxO1, and IL-10 (Figure 4G, top right) protein expressions are associated with M2 macrophage anti-inflammatory functions, reflecting immune dysregulation during retroviral infection. Conversely, CD169 is reflective of activated macrophages, particularly regarding foreign antigen capture for presentation. Taken together, our results indicate that both phenotypic composition and functional marker expression patterns may be altered due to viral infection, with the potential for immune functional dysregulation.

### Tissue architecture is remodeled during viral infection

**Figure 5:**
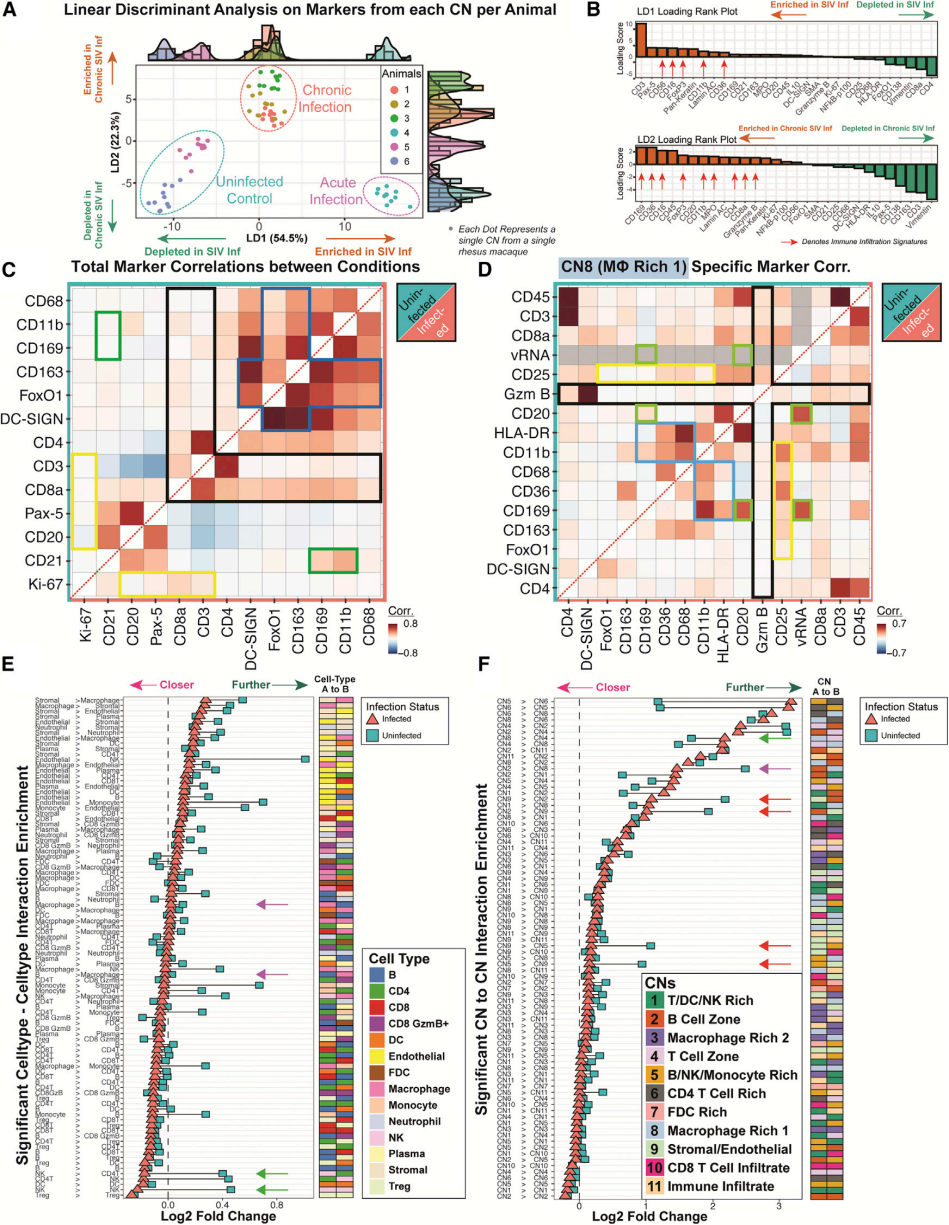
Observation of SIV-specific cellular responses and tissue reorganization (A) LDA was performed on the collective markers for each of the 14 CNs within each of the six animals. Each dot represents a single CN from a single animal. CNs are colored by the animal infection status and the animal of origin. Dotted lines indicate animal infection status: uninfected control (cyan), acute infection (purple), and chronic infection (orange). The variances accounted for by each of the two linear discriminants, LD1 and LD2, are plotted on the x and y axes, respectively. (B) The rank plot of the markers that account for LD1 (top) and LD2 (bottom) colored based on enrichment (orange) or depletion (green) in individual CNs of infected animals versus uninfected animals. (C) Pairwise Pearson’s correlations of selected immune markers across each individual cell from healthy (top; teal) and SIV-infected (bottom; orange) animals. Representative infection-related processes are highlighted: (1) macrophage immunosuppression as indicated by a M2 switch via CD163 and FoxO1 (blue boxes), (2) increased CD8 T cell infiltration (black boxes), (3) B and T cell proliferation via elevated Ki-67 correlation (yellow boxes), and (4) FDC activation and antigen presentation via increased CD169 and CD11b presence (green boxes). (D) Pairwise Pearson’s correlations of selected immune markers across each individual cell within CN8, separated by healthy (top; teal) and SIV-infected (bottom; orange) animals. The colored boxes are as above in (C). (E and F) The pairwise cell distances for (E) each cell type and (F) each CN over 1,000 iterations of randomized background permutations are plotted as colored shapes for infected (orange, triangle) and healthy (teal, square) animals. Only interactions that passed a correlation test (p < 0.05) for both infection conditions are shown. Shapes that are toward the left indicate cell-cell or CN-CN interactions that are closer than expected, and those toward the right indicate interactions that are further apart than expected. Pairs of cells are given in text form (left) and colored heatmaps (right). In (E), purple arrows indicate B cell-macrophage interactions, and green arrows indicate NK cell-T cell interactions that are closer in infected than uninfected tissues. In (F), the green arrow indicates CN8-CN4 interactions that are closer in uninfected than infected tissues, whereas purple and red arrows indicate interactions that are closer in infected tissues than uninfected tissues. See also Figure S5.

We postulated that CNs can recapitulate the underlying tissue biology using both its cell type composition and functional marker quantifications. We performed linear discriminant analysis (LDA) on the accumulated marker compositions within each CN from each animal (6 animals with 11 CNs from each). LDA analysis separated CNs from infected and uninfected rhesus macaques (Figure 5A, left) and further stratified the animals by chronic versus acute viral infection status (Figure 5A, right). LD1, which accounted for 54.5% of the variation, separated infected and uninfected animals and their associated CNs (Figures 5A and 5B, top). LD2, which captured 22.3% of the variation, distinguished between chronic and nonchronic infection statuses (Figures 5A and 5B, bottom). Factors differentiating SIV-infected from SIV-uninfected animals included CD56, CD16, FoxP3, CD11b, and CD36. Factors differentiating SIV chronic from nonchronic status included CD169, CD36, CD16, FoxP3, CD11b, MPO, CD4, CD8a, and granzyme B (Figure 5B).

Using co-occurrences of markers as a proxy to understand global tissue reorganization triggered by viral infection, we calculated the Pearson’s correlations between marker pairs for SIV-negative (Figure 5C, top; teal) and SIV-positive (Figure 5C, bottom; orange) conditions. We focused on markers of cell types dysregulated during SIV infection such as those that characterize B cells, T cells, and macrophages. In agreement with previous data, our strategy highlighted the following infection-driven processes: (1) macrophage immunosuppression as indicated by a M2 switch via CD163 and FoxO1 (blue boxes), (2) increased CD8 T cell infiltration (black boxes), (3) B and T cell proliferation via elevated Ki-67 correlation (yellow boxes), and (4) macrophage/FDC activation and function via increased CD169 and CD11b presence (green boxes) (Figure 5C).

Specific microenvironment interactions were also apparent when Pearson’s correlations between the marker pairs within each CN were analyzed (Figure S5A). Notably, for the macrophage-rich CN8 within SIV-positive tissues, there was evidence of (1) increased CD169 expression but decreased HLA-DR (blue boxes), (2) decreased granzyme B activity (black boxes), (3) increased CD25 correlation (yellow boxes), and (4) elevated B cell association with vRNA and CD169 expression (green boxes) (Figure 5D). The pairwise marker correlation maps from single cells in each infection condition and CN provide an informed view of dysregulation and reorganization induced in response to viral infection (Figures 5C, 5D, and S5A).

To understand how cells are functionally positioned differently between healthy and infected microenvironments, we compared the direction-specific, cell-cell pairwise interactions for each FOV against a randomized background model (Figure S5B). We first identified tissue interactions that were either closer (Figure 5E, magenta arrow pointing left) or further (Figure 5E, green arrow pointing right) relative to the background in tissues. Interaction enrichments were then ranked by the SIV-infected status for visualization purposes. We observed an increase in both NK-CD4 T cell and NK-NK cell interactions (Figure 5E, green arrows), and B cell-macrophage and macrophage-B cell interactions upon infection (Figure 5E, purple arrows).

We next investigated how CNs were modulated upon viral infection using a direction-specific CN-CN pairwise interaction enrichment analysis over a random background model (Figure 5F). Interactions involving blood vessel-enriched CN9 with CN2 (B cell zone) and with CN5 (B cell-, NK cell-, and monocyte-rich) were prominent in infected tissues (Figure 5F, red arrows), demonstrating increased endothelial interactions and physical proximity of immune cells to specific microenvironments within infected tissues. We also observed that interactions between macrophage-enriched CN8 with CN4 (T cell zone) were decreased (Figure 5F, green arrow), whereas interactions between B cell zone-associated CN2 with macrophage-rich CN8 were increased in infected tissues (Figure 5F, purple arrow). The latter observation agrees with the detected increase above in B cell-macrophage and macrophage-B cell interactions (Figure 5E, purple arrows), although they did not physically overlap (Figure S4B). These analyses show functional tissue remodeling during viral infection.

### B cells and macrophages are associated with IL-10-induced immunosuppressive microenvironments

**Figure 6:**
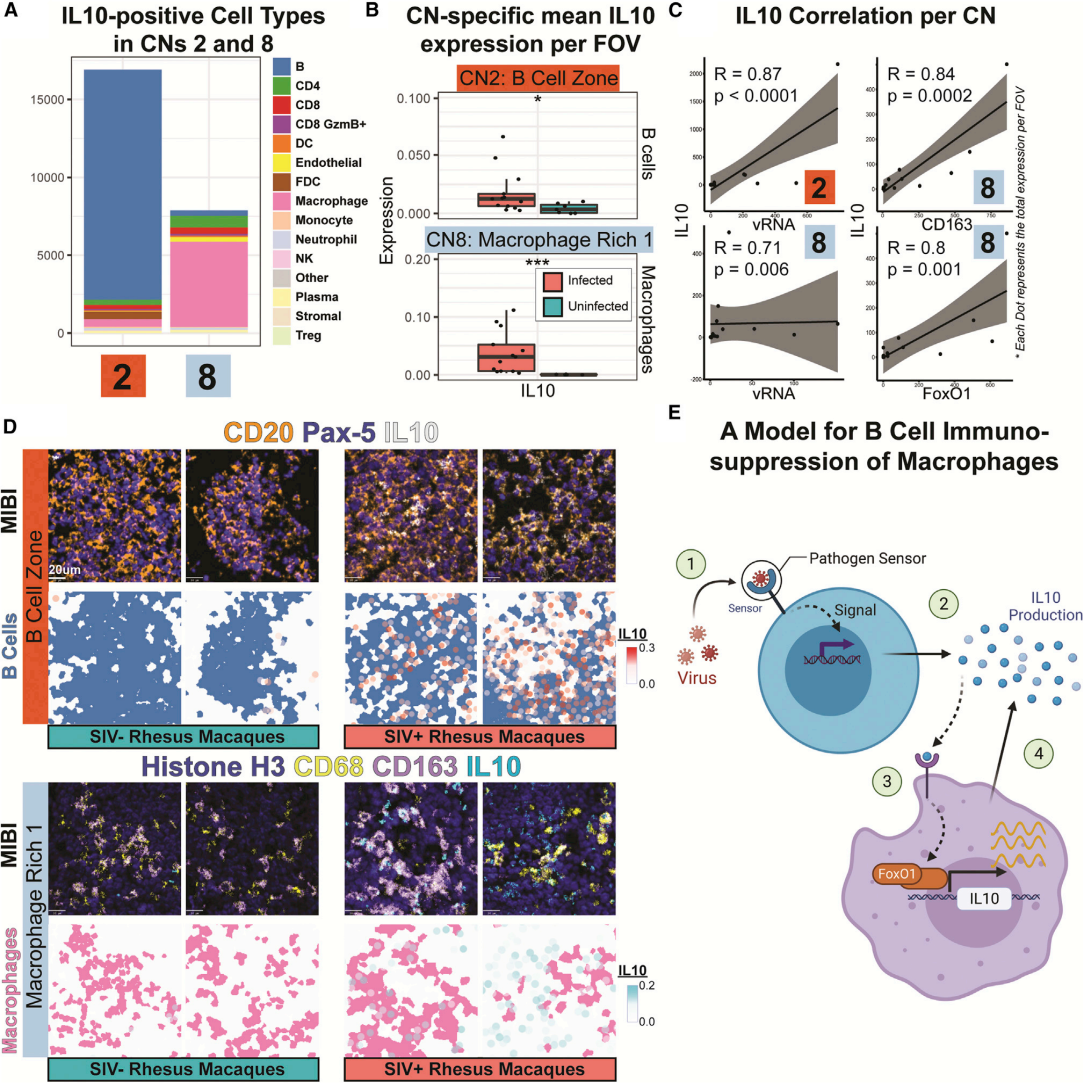
B cell-driven IL-10 production is correlated with macrophage polarization and immunosuppression during SIV infection (A) Bar plot of numbers of IL-10-positive cells of the indicated types in CN2 and CN8 in all animals. (B) Box plots of mean IL-10 expression across B cells within the CN2 and across macrophages in CN8. Each dot represents data from a single FOV from SIV-infected and SIV-uninfected controls. Nonpaired Wilcoxon test: ns, not significant; ^∗^p < 0.05; ^∗∗^p < 0.01; ^∗∗∗^p < 0.001. (C) Plots of Spearman’s correlations between IL-10 quantities and vRNA in CN2 (top left), and CN8 (bottom left), vRNA with the M2 immunosuppressive macrophage marker CD163 in CN8 (top right), and FoxO1 in CN8 (bottom right). Each dot represents data from a single SIV-infected FOV. (D) Representative pseudocolored MIBI images depicting IL-10, B cell markers (CD20 and Pax-5; top), and macrophage markers (CD68 and CD163; bottom). Two representative FOVs from infected and uninfected animals are shown. The phenotype maps superimposed with IL-10 expression patterns are shown below each MIBI image. (E) A cartoon depicting a proposed model for B cell-induced immunosuppression of macrophages via IL-10. See also Figure S6.

Our results suggest a strong linkage between B cells and macrophages during SIV infection (Figure 5E, purple arrows), specifically between CN2 (B cell zone) and CN8 (macrophage rich 1) (Figure 5F, purple arrow). The IL-10 expression patterns were distinctive in these neighborhoods: cells positive for IL-10 were predominantly B cells in CN2 (Figure 6A, left) and were predominantly macrophages in CN8 (Figure 6A, right). IL-10 is an immunoregulatory cytokine that can activate or suppress the immune system (Ouyang and O’Garra, 2019; Rojas et al., 2017). IL-10 expression is upregulated in patients with HIV within several circulating immune cell types, including B cells (Brockman et al., 2009), and in lymphoid tissues after SIV infection (Estes et al., 2006; Tabb et al., 2013). We observed this increase in both B cells in CN2 and macrophages in CN8 after SIV infection (Figure 6B), implicating elevated IL-10 expression in these cells as a host response to viral infection. We found a positive correlation between vRNA and IL-10 within CN2 (Figure 6C, top left panel; R = 0.87 and p < 0.0001) and CN8 (Figure 6C, bottom left panel; R = 0.71 and p = 0.006). This correlation held true even after excluding the acutely infected outlier (Figures S6A and S6B). Subsampling 75% of the data over 500 iterations also showed the robustness of this positive correlation between IL-10 and vRNA in CN2 and CN8 (Figures S6C and S6D). These results suggest IL-10 secretion by B cells as a key correlate regarding tissue responses to viral presence within the lymph nodes.

In line with the immunosuppressive potential of IL-10, we observed a positive correlation between IL10 and immunosuppressive M2 macrophage markers, CD163 and FoxO1, in CN8 (Figure 6C, right panels). We visually confirmed these findings on upregulation of IL-10 within B cells (Figure 6D, top) and CD163^+^ M2 macrophages (Figure 6D, bottom) in response to SIV infection. Together, these results support a role for how SIV infection can suppress host tissue immune responses, possibly through (1) initial sensing of viral particles by B cells via innate antiviral sensors, (2) production of IL-10, potentially by B regulatory cells, that attracts nearby macrophages (Glass et al., 2021; Figure 5F), (3) subsequent FoxO1 activation that leads to more IL-10 production (Chung et al., 2015), and finally (4) M2 macrophage differentiation and creation of an immunosuppressive TME (Rojas et al., 2017; Figure 6E).

### Tissue environmental cues can influence SIV viral transcription state

**Figure 7:**
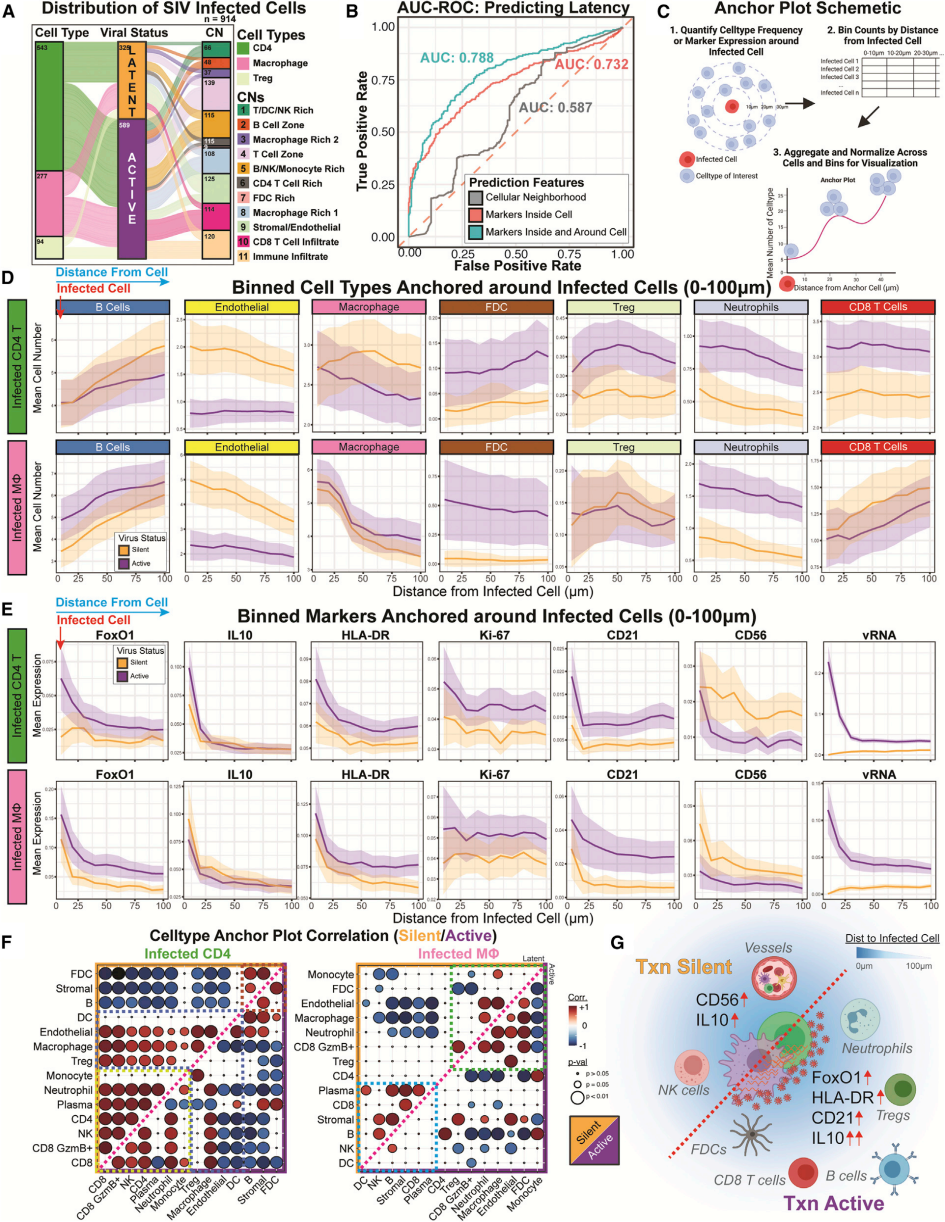
Spatial interrogation of tissue microenvironmental determinants for retroviral transcription state (A) An alluvial plot depicting the compositions of SIV-infected cell types (CD4^+^ T cells, n = 543; macrophages, n = 277; and Tregs, n = 94), their viral transcription status, and their associated CNs. (B) Predictive performances of classifiers (1) CNs (gray, AUC = 0.587), (2) markers inside the infected cell (orange, AUC = 0.732), and (3) markers from a cell and its nearest neighbors (teal, AUC = 0.788). The dotted-red line indicates AUC of 0.5, as expected by chance. (C) A schematic depicting how the anchor plots were calculated for the anchor plots in (D) and (E). In short, (1) mean cell type frequencies or marker expressions around each infected cell were tabulated, (2) these values were binned by their distance from the infected cell in 10-μm increments, and (3) data for all infected cells were aggregated and normalized for visualization. (D and E) Anchor plots of (D) mean cell type quantifications and (E) mean marker expression around infected CD4^+^ T cells (top) or macrophages (bottom). Orange indicates transcriptionally silent cells, and purple indicates actively transcribing cells. The thick colored lines represent the means, and light regions around these lines depict the 95% confidence intervals. (F) Heatmaps of Pearson’s correlations for cell type pairs for infected CD4^+^ T cells (left) and macrophages (right). Transcriptionally silent infection correlation heatmaps are represented in the top left (orange) and active infection correlation heatmaps are in the bottom right (purple). The sizes of the circles reflect the p values from the correlation test for association, and colors indicate degree of correlation. (G) Schematic representing the tissue correlates and determinants of retroviral transcription status in retroviral reservoirs. See also Figure S7.

Codetection of vDNA, vRNA, and host proteins enabled by PANINI is particularly suitable for differentiating SIV-infected cells that are transcriptionally silent (i.e., vDNA^+^ and vRNA^−^) or active (vDNA^+^ and vRNA^+^). We identified 914 SIV-infected cells within rhesus macaque lymph nodes, comprising of CD4^+^ T cells (69.7% of which 10.3% were Tregs) or macrophages (30.3%) (Figure 7A). Consistent with the viremic nature of these animals, the infected cells were predominately transcriptionally active (64.4%), with a similar composition of transcription status within each cell type and CN of origin (Figure 7A), except for the higher presence of transcriptionally silent cells within the stromal and endothelial rich CN9 (Figure 7A).

To identify cellular and CN features predictive of viral transcription state, we trained a random forest classifier on (1) CN information alone, (2) cell marker features within the infected cell, and (3) cell marker features within the 20-cell-radius (i.e., 19 neighbors and the infected cell). Viral RNA and NF-κB-p100 were excluded from this analysis (Hiscott et al., 2001). We observed that CNs alone were poor predictors of viral transcription state, with the area under the curve (AUC) of the receiver operating characteristics curve at 0.587, close to what is expected by chance (Figure 7B). Markers present on or within infected cells were better predictors of viral transcription state than CNs (AUC: 0.732), whereas utilizing the combined markers of the infected cell and its 19 nearest neighbors (i.e., markers within the CN group) achieved the highest performance (AUC: 0.788). These observations indicate that factors both intrinsic to the infected cell and those from the environment influence viral transcription status.

How cells communicate through cell-to-cell interactions and soluble mediators are products of their proximity to each other and the marker expression patterns in their vicinity. We devised a meta-analytical method to quantify cell types and their marker expressions within a 100-μm radius around aggregated infected cells (Figure 7C). We focused on the more abundant non-Treg-infected CD4^+^ T cells and macrophages, given the low number of infected Tregs (n = 94) (Figure 7A). We observed that infected cells tended to be within regions with high B cell density independent of transcription status (Figure 7D). Transcriptionally silent infected cells were generally closer to endothelial cells than transcriptionally active cells (Figure 7D). Infected cells were also in proximity to macrophages, although this did not depend on viral transcription status or type of infected cell (Figure 7D). We observed higher densities of FDCs, Tregs, neutrophils, and CD8^+^ T cells in the vicinities of infected CD4^+^ T cells actively producing vRNA than in the vicinities of transcriptionally silent CD4^+^ T cells (Figure 7D). This was also observed for transcriptionally active infected macrophages, except for proximal Tregs and CD8^+^ T cells (Figure 7D).

Quantifying functional markers enables insights into the microenvironment beyond cell phenotypes. vRNA expression patterns around cells actively transcribing vRNA followed a point spread pattern from the source (Figure 7E, right). We noted a stark scaling of the amount of FoxO1 expression as a function of distance from transcriptionally active versus silent cells (Figure 7E). IL-10 tended to be elevated around all infected cells, and were higher around transcriptionally active versus silent CD4^+^ T cells (Figure 7E). Similar trends were observed for HLA-DR, Ki-67, and CD21, where higher proximal expressions correlated with viral activation within infected cells (Figure 7E). The reverse was observed for CD56, where elevation around transcriptionally latent infected cells suggested a role of NK cells in controlling viral transcription (Figure 7E). We also detected similar and distinctive patterns for various markers in infected CD4^+^ T cells and macrophages (Figures 7D, 7E, and S7), emphasizing the role of both the cellular phenotype and functional markers around infected cells in viral transcription status (Figures 7D, 7E, and S7).

### Identification of spatial correlates and tissue microenvironmental immune responses during infection

To better interpret the complexity of orchestrated tissue events around SIV-infected cells, we computed the Pearson’s correlations between each pair of cell type frequencies as a function of distance from the infected cell (Figure 7F; top, yellow: transcriptionally silent cells, bottom, purple: active cells). Three modules were detected in infected CD4^+^ T cells (Figure 7F, left). One module of interaction involving FDCs, stromal cells, and B cells, factors essential for germinal center functions, was disrupted during viral RNA transcription in infected CD4^+^ T cells (Figure 7F, left). Another module, populated by dendritic cells, endothelial cells, macrophages, and Tregs, was anticorrelated in active infected CD4^+^ T cells (Figure 7F, left). The third module, composed of monocytes, neutrophils, plasma cells, CD4^+^ T cells, NK, and CD8^+^ T cells, differentiated CD4^+^ T cell viral transcription status (Figure 7F, left). For infected macrophages, we observed two distinct signature modules (Figure 7F, right). The first module was dominated by monocytes, FDCs, macrophages, neutrophils, endothelial, CD8^+^ granzyme B^+^ cells, and Tregs. The second involved plasma cells, CD8^+^ T cells, stromal cells, B cells, NK cells, and dendritic cells. Taken together, these plots highlight an interplay between virus infection, transcription, and microenvironmental proximity as effectors or consequences of viral infections. Our data suggest a probable model in which the distances of both functional markers (e.g., CD56, IL-10, FoxO1, HLA-DR, and CD21) and cell types (e.g., endothelial vessels, NK, FDCs, neutrophils, Tregs, B cells, and CD8^+^ T cells) from SIV-infected cells can both influence or be influenced by viral transcription status (Figure 7G).

## Discussion

Here, we describe the development and validation of PANINI, a framework that enables detection of low-copy nucleic acids while preserving confident detection of protein epitopes. This was achieved through a combination of heat-induced epitope retrieval in a pH9 buffer, protease-free branched-chain amplification of nucleic acids, tyramide signal amplification coupled with hapten deposition, followed by multiplexed antibody imaging. We then coupled PANINI with the MIBI to visualize integrated vDNA and vRNA in SIV-infected cells and viral particles, along with 31 immune phenotypic and functional protein markers (Figure S1). We demonstrate the utility of PANINI in the detection of nucleic acid copies down to single events in archival FFPE tissues to interrogate the diverse immune responses within SIV-infected lymphoid tissues.

We first confirm hallmarks of retroviral infection, including CD4^+^ T cell depletion (Figures 3C and 3D; Hazenberg et al., 2000), a heightened NK cell and CD8^+^ T cell response (Figure 3E; Alter et al., 2007; Goonetilleke et al., 2009), and a lack of immune infiltration into the “sanctuary” B cell follicles (Figures 4B and 4E; Fukazawa et al., 2015). By integrating CN information with the markers that are present within them, we were able to segregate between infection status using LDA (Figure 5A).

The role and distribution of IL-10 in tissues is largely unclear (Brockman et al., 2009; Estes et al., 2006; Fukazawa et al., 2015; Tabb et al., 2013). Here, we reveal a B cell response to SIV infection through the secretion of IL-10, possibly in addition to other cytokines. This is correlated to the attraction and immunosuppression of macrophages in its vicinity, via an infection-driven M2-phenotypic switch, highlighting a route for immunoregulation of SIV-infected tissues (Figure 6). Such a dampened environment, with heightened IL-10, FoxO1, and HLA-DR expression 20–30 μm around infected cells, may in part explain viral transcription status (Figure 7). Emerging orthogonal evidence in increased IL-10 protein expression and transcriptomic signatures during chronic and ART-treated retrovirus infections are compelling (Harper et al., 2022; Ribeiro et al., 2021). This is just one example of the temporal ordering of distinctive tissue features during SIV infection that warrant further investigation stemming from this work.

PANINI is particularly suited for disentangling environmental effects from intrinsic properties of the cell. We demonstrated this using SIV-infected rhesus macaques as a model, with a particular focus on viral reservoirs within lymphoid tissues, sites previously described to be a primary location of infected cells (Estes et al., 2017). We confirmed FDCs as the largest repository of vRNA (i.e., viral particle deposition) during viremic SIV infection, whereas CD4^+^ T cells and macrophages are the primary cell types infected. We then uncovered both extrinsic and intrinsic features that best predict viral activation status. Additional contributing features that distinguish between viral transcription activity include the expression of CD56 and quantities of Tregs, neutrophils, and CD8 T cells (Figure 7G).

The establishment of the PANINI experimental platform, a 33-marker panel compatible with FFPE archival tissues, spatial analytical workflow, and conceptual framework for multimodal analysis of tissue features, has enabled reinterrogation of previous observations and establishment of new models and hypotheses. Properly designed and executed antibody-based multiplexed imaging has distinctive advantages, including (1) robust readouts with large dynamic ranges, and (2) assessment of molecular pathways through the measurement of key protein mediators of cellular function and identity. Readers are referred to recent resources that show high concordance between single-plex immunohistochemistry and MIBI-TOF imaging (Liu et al., 2022) and a practical guide to selecting and validating antibodies for high-dimensional imaging modalities (Hickey et al., 2021).

Fundamental questions remain, including: (1) how do CNs and infected cells change with antiretroviral therapy (ART) or immunotherapy? (2) Are features and relationships different in other tissue sites, such as the brain- or gut-associated lymphoid tissue? (3) Can these principles be translated to other infectious diseases such as tumor virus-driven malignancies, SARS-CoV-2, tuberculosis, or cancer biology questions involving copy-number amplifications, repetitive elements, and extrachromosomal DNA? Future work to combine antibody-based protein spatial omics with other platforms capable of measuring other biomolecules at scale *in situ* will be paramount to improve our understanding of viral infections (Merritt et al., 2020; Sun et al., 2021). We anticipate that PANINI, coupled with widely adopted multiplexed imaging technologies, validated nucleic acid probes and antibodies, and robust animal models or archival clinical samples, will be a step toward for advancing the mechanistic insights needed to better guide therapeutic intervention strategies.

### Limitations of the study

It is of note that a fraction of vDNA-positive cells in SIV-infected lymph nodes were macrophages (Figure 7A). The static nature of imaging archival tissues, coupled with the lower image resolutions acquired in this study limit our ability to distinguish between productively infected macrophages and infected CD4^+^ T cells that were phagocytosed. Additionally, our study consisted of 33 markers (2 nucleic acid and 31 proteins) due to low antibody cross-compatibility between human and nonhuman primate samples. Further efforts aimed at developing and validating nonhuman primate-specific reagents are underway (Jiang et al., 2021; McCaffrey et al., 2022). The incorporation of SIV proteins, although not performed in this study, will be essential in further functional stratification of infection status. Finally, the limited cohort size (six rhesus macaques, 20 field of views, and >400,000 cells) encumbered further biological insights into SIV pathogenesis in rhesus macaques. In-depth analysis of larger cohorts with temporal sampling are underway to extend upon the findings in this study.

## STAR★Methods

### Key resources table

**Table:** 

REAGENT or RESOURCE	SOURCE	IDENTIFIER
**Antibodies**		

dsDNA (Clone 35I9 DNA)	Abcam	Clone ID: 35I9 Cat #:ab27156
Vimentin (Clone D21H3)	Cell Signaling Technology	Clone ID: D21H3 Custom Order for Carrier Free
Histone H3 (Clone D1H2)	Cell Signaling Technology	Clone ID: D1H2 Custom Order for Carrier Free
CD16 (Clone D1N9L)	Cell Signaling Technology	Clone ID: D1N9L Custom Order for Carrier Free
SMA (Clone D4K9N)	Cell Signaling Technology	Clone ID: D4K9N Custom Order for Carrier Free
CD209 (DC-SIGN) (Clone DCN46)	Biolegend	Clone ID: DCN46 Cat #: 551186
NFkB-p100 (pS865) (Polyclonal)	Abcam	Cat #: ab31474
CD4 (Clone EPR6855)	Abcam	Clone ID: EPR6855 Cat #: ab181724
CD56 (Clone MRQ-42)	Cell Marque	Clone ID: MRQ-42 Custom Order for Carrier Free
FoxP3 (Clone 236A/E7)	Thermo Fischer Scientific	Clone ID: 236A/E7 Cat #: 14-4777-82
Granzyme B (Clone EPR20129-217)	Abcam	Clone ID: EPR20129-217 Cat #: ab219803
CD21 (CR2) (Clone SP186)	Abcam	Clone ID: SP186 Cat #: ab240987
Ki-67 (Clone 8D5)	Cell Signaling Technology	Clone ID: 8D5 Custom Order for Carrier Free
Pax-5 (Clone D7H5X)	Cell Signaling Technology	Clone ID: D7H5X Custom Order for Carrier Free
CD138 (Clone EPR6454)	Abcam	Clone ID: EPR6454 Cat #: ab226108
CD163 (Clone EDHu-1)	Novus	Clone ID: EDHu-1 Cat #: NB110-40686
CD68 (Clone D4B9C)	Cell Signaling Technology	Clone ID: D4B9C Custom Order for Carrier Free
FoxO1 (Clone C29H4)	Cell Signaling Technology	Clone ID: C29H4 Custom Order for Carrier Free
CD3 (Clone MRQ-39)	Cell Marque	Clone ID: MRQ-39 Custom Order for Carrier Free
CD20 (Clone SP32)	Abcam	Clone ID: SP32 Cat #: ab64088
Lamin A/C (Clone EPR4100)	Abcam	Clone ID: EPR4100 Cat #: ab216074
MPO (Polyclonal)	R&D Systems	Cat #: AF3667
HLA-DR (Clone EPR3692)	Abcam	Clone ID: EPR3692 Cat #: ab215985
IL-10 (Clone 4A7-25-17)	Abcam	Clone ID: 4A7-25-17 Cat #: ab134742
CD169 (Clone SP213)	Abcam	Clone ID: SP213 Cat #: ab245735
CD8a (Clone D8A8Y)	Cell Signaling Technology	Clone ID: D8A8Y Custom Order for Carrier Free
Pan-Keratin (Clone AE1/AE3)	Biolegend	Clone ID: AE1/AE3 Cat #: 914204
CD11b (Clone EPR1344)	Abcam	Clone ID: EPR1344 Cat #: ab209970
CD36 (Clone D8L9T)	Cell Signaling Technology	Clone ID: D8L9T Custom Order for Carrier Free
CD45 (Clone D9M8I)	Cell Signaling Technology	Clone ID: D9M8I Custom Order for Carrier Free
Anti-Biotin (Clone 1D4-C5)	Biolegend	Clone ID: 1D4-C5 Cat #: 409002
Anti-Digoxigenin (Clone 21H8)	Abcam	Clone ID: 21H8 Cat #: ab420
CD15 (Clone MMA)	BDBiosciences	Clone ID: MMA Cat #: 559045
MUC1 (Clone MUC1/955)	NSJ Bioreagents	Clone ID: MUC1/955 Cat #: V2372SAF

**Bacterial and virus strains**		

SIVmac251	AIDS reagent resource	Cat #: 253
SIVmac239	Mudd et al., 2018; PMID: 30262807	N/A

**Biological samples**		

FFPE Inguinal LN from acute SIVmac239 infected rhesus macaque (Day 13 p.i.)	Mudd et al., 2018; PMID: 30262807	Animal ID: RHCF4T
FFPE Mesenteric LN from chronic SIVmac239X infected rhesus macaque (week 16 p.i.)	Oregon National Primate Research Laboratory	Animal ID: 34675
FFPE Mesenteric LN from chronic SIVmac239X infected rhesus macaque (week 19 p.i.)	Oregon National Primate Research Laboratory	Animal ID: 34622
FFPE Mesenteric LN from chronic SIVmac251 infected rhesus macaque (Day 227 p.i.)	Oregon National Primate Research Laboratory	Animal ID: 33098
FFPE Mesenteric LN from SIV neg rhesus macaque	Oregon National Primate Research Laboratory	Animal ID: 32518
FFPE Inguinal LN from SIV neg rhesus macaque	NCI/ACVP	Animal ID: A7E033A

**Chemicals, peptides, and recombinant proteins**		

TBS IHC Wash Buffer plus Tween 20	Sigma	Cat #: 935B-09
Dako Target Retrieval Solution, pH 9	Agilent	Cat #: S236784-2
Dako Target Retrieval Solution, pH 9	Agilent	Cat #: S236784-2
Avidin/Biotin Blocking Kit	Biolegend	Cat #: 927301
Glutaraldehyde 10% Aqueous Solution EM Grade	EMS	Cat #: 16120
Donkey Serum	Sigma	Cat #: D9663-10ML
16% Paraformaldehyde (formaldehyde) aqueous solution	EMS	Cat #: 15711
VECTABOND Reagent for Tissue Section Adhesion	Vector Labs	Cat #: SP-1800
TCEP	Sigma	Cat #: C4706-10G
Candor PBS antibody stabilizer	Fisher Scientific	Cat #: NC0436689

**Critical commercial assays**		

RNAscope Multiplex Fluorescent Detection Kit V2	Biotechne	Cat #: 323110
RNAscope v2.5 HD Detection Brown	Biotechne	Cat #: 322310
SIVmac239-gag-pol sense (vDNA) probe	Biotechne	Cat #: 416141
SIVmac239-vif-env-nef-tar (vRNA) probe	Biotechne	Cat #: 416131-C2
Human CCL2 probe	Biotechne	Cat #: 423811
Human IL8 probe	Biotechne	Cat #: 310381-C2
Maxpar X8 Multimetal Labeling Kit	Fluidigm	Cat #: 201300
Ionpath Conjugation Kits	Ionpath	Cat #: 600XXX
TSA Plus Biotin 50-150 slides	Akoya	Cat #: NEL749A001KT
TSA Plus DIG, 50-150 Slides	Akoya	Cat #: NEL748001KT

**Deposited data**		

Multiplexed Images	This Study	Data available at: www.mibi-share.ionpath.com

**Experimental models: Cell lines**		

3D8	AIDS reagent resource	13239
174XCEM	AIDS reagent resource	272

**Experimental models: Organisms/strains**		

Rhesus macaques of Indian origin	ONPRC/NCI	N/A

**Software and algorithms**		

Matlab 2019b	Mathworks	N/A
MIBIAnalysis	Keren et al., 2018	https://github.com/lkeren/MIBIAnalysis
R 3.6.3	R Core Team, 2020	https://www.r-project.org/
Cellular Neighborhoods	Schürch et al., 2020	https://github.com/nolanlab/NeighborhoodCoordination
DeepCell 0.6.0	Greenwald et al., 2021	https://github.com/vanvalenlab/deepcell-tf
Mesmer	Greenwald et al., 2021	https://github.com/vanvalenlab/deepcell-tf
CellEngine	Primity Bio	https://cellengine.com/
FlowSOM	Van Gassen et al., 2015	https://bioconductor.org/packages/release/bioc/html/FlowSOM.html
MEM	Diggins et al., 2017	https://github.com/cytolab/mem
Hmisc R package	N/A	https://cran.r-project.org/web/packages/Hmisc/index.html
MASS R package	Venables and Ripley, 2002	https://cran.r-project.org/web/packages/MASS/index.html
deldir R package	N/A	https://cran.r-project.org/web/packages/deldir/index.html
caret R package	N/A	https://cran.r-project.org/web/packages/caret/index.html
ggplots2 R package	N/A	https://cran.r-project.org/web/packages/ggplot2/index.html
MIBITracker	N/A	https://mibi-share.ionpath.com/
Code related to this manuscript	This Study	https://doi.org/10.5281/zenodo.6381128

**Other**		

MIBIscope (Alpha Iteration)	Ionpath	N/A
ACD HybEZ Hybridization System	Biotechne	310013
Lab Vision PT Module	Fisher Scientific	A80400012

### Resource availability

#### Lead contact

Further information and requests for resources and reagents should be directed to and will be fulfilled by corresponding author, and lead contact, Garry Nolan (gnolan@stanford.edu).

#### Materials availability

This study did not generate new unique reagents.

### Experimental model and subject details

#### Animal experiments and tissue acquisition

Archival FFPE tissues were obtained from SIV-infected and control rhesus macaques (Macaca mulatta) of Indian origin that were housed at the Oregon National Primate Research Center (OR, USA) and at the National Institutes of Health (Bethesda, MD, USA) with the approval of the respective Institutional Animal Care and Use Committees. The animal experiments were conducted with strict adherence to the NIH and the Animal Welfare Act and in accordance with American Association for the Accreditation of Laboratory Animal Care (AAALAC) standards in AAALAC-accredited facilities.

Our cohort consisted of the following animals: SIV-negative (n=2) and SIV-challenged (13 day or 16–19 wpi; n=4). Lymph nodes were collected at necropsy and immediately fixed in freshly prepared neutral buffered 4% PFA for 24 hours at room temperature. Afterwards, the fixative was replaced with 80% ethanol and the tissues were processed through a series of 30-minute incubations in increasing alcohol concentrations to 100 percent, then in xylene and hot paraffin, in a Tissue Tek Vacuum Infiltration Processor 6 (Sakura). Processed tissues were then paraffin embedded and stored in a cool, dry place. All animal, virologic, age, sex and tissue data are summarized in Table S1.

#### COVID-19 Tissue Specimen Collection

Human SARS-CoV-2 infection and control lung tissues were obtained during autopsy at the University Hospital Basel Switzerland. The use of SARS-CoV-2 infected tissue was approved by the ethics commission of Northern Switzerland (EKNZ; study ID: 2020-00969). All COVID-19 patients or their relatives consented to the use of tissue for research purposes.

### Method details

#### Antibody Conjugation

Antibodies were conjugated to metal polymers using the Maxpar X8 Multimetal Labeling Kit (Fluidigm, 201300) and Ionpath Conjugation Kits (Ionpath, 600XXX) with slight modifications to manufacturer protocols. The antibodies used and their respective clones are listed in the key resources table. Antibody conjugation was performed exactly as described previously (Han et al., 2018). In short, 100ug of carrier free antibodies are subject to gentle reduction in the presence of 4uM of TCEP for 30 min, before conjugation to lanthanide-loaded polymers. Post elution, all antibodies are quantified via nanodrop (Thermo Fisher Scientific, ND2000), diluted with >30% w/v Candor PBS antibody stabilizer containing 0.02% w/v NaN3 (Thermo Fisher Scientific, nc0436689) and stored at 4°C until use.

#### Antibody panel titration and validation

The antibody panel targets were selected based on their ability to delineate specific cell-types or lineages (e.g. CD4), or have a functional readout (e.g. NF-kB p100). FFPE-compatible antibody clones that had worked in our laboratories previously were collated, with additional sourcing of antibody clones from the literature. These sourced clones were tested via traditional IHC to ensure compatibility and robustness of staining with the PANINI buffer conditions (see below). Working antibody clones were then conjugated as described above and underwent further titration and validation on the MIBI platform to assess for staining specificity. We generally start with a working concentration of 1ug/ml for titrations and go up or down three-fold in serial sections that are stained concurrently with antibodies diluted from the same master mix. We evaluated for staining intensity, patterns, co-staining with other markers (e.g. CD3 with CD4) and potential signal spillover in the hydride (+1), oxide (+16) and hydroxide (+17) ion channels (Chevrier et al., 2018; Keren et al., 2018). Antibodies that did not retain binding capabilities after the conjugation were discarded. The final targets, metal tags and optimized antibody titers used in this study are described in Table S2. This detailed panel and titers are also deposited, along with the study images, on https://mibi-share.ionpath.com/. For a more detailed guide on antibody target selection and optimization, as well as IHC and MIBI cross comparisons, readers are referred to the following resources (Hickey et al., 2021, Liu et al., 2022).

#### Gold slide preparation

Gold slides were prepared as previously described (Ji et al., 2020; Keren et al., 2018). Briefly, Superfrost Plus glass slides (Thermo Fisher Scientific, #12-550-15) were soaked in dish detergent, rinsed with distilled water followed by acetone. Acetone evaporation was performed under a constant stream of air in a fume hood, and clean slides subsequently coated with 30nm of Tantalum followed by 100nm of Gold at the Stanford Nano Shared Facility (SNSF; Stanford CA) and New Wave Thin Films (Newark, CA).

#### Vectabond Pre-treatment of Gold Slides

Gold slides were pretreated with Vectabond (Vector Labs, #SP-1800) according to the manufacturer’s protocols. In short, slides were submerged in 100% acetone for 5 min before incubation in a glass beaker containing a mixture of 2.5 ml Vectabond and 125 ml 100% acetone for 30 min. Slides were then washed in 100% acetone for 30 sec, air dried, and stored at room temperature.

#### Cell Culture and FFPE Cell Pellet Embedding

The well-characterized SIV-infected cell line 3D8, which contains a single integrated provirus per cell (Mattapallil et al., 2005), and the uninfected parental 174xCEM cell line were used to validate our detection of vRNA and vDNA as part of the PANINI workflow (see below). Cell were fixed in 4% paraformaldehyde (PFA) overnight before embedding into Histogel (Fisher Scientific, NC9150318) and paraffin wax as described previously (Deleage et al., 2016).

#### RNAScope & DNAScope Fluorescent Multiplex *in situ* Hybridization

The RNAScope & DNAScope multiplex staining methodology originally described in Deleage et al. (2016) was modified and optimized to increase the feasibility of using a pH9 antigen retrieval condition without protease digestion to detect both SIV vDNA and vRNA. FFPE sections of SIV-positive and SIV-negative rhesus macaque lymph nodes on Fisher Superfrost glass microscopic slides were deparaffinized by heating at 60°C for 1h and then transferred to a xylene bath for 5 mins. Slides were transferred to a new xylene bath for another 5 min, followed by 2 x 1 min incubations in 100% EtOH baths. Slides were then rinsed with double distilled water (ddH2O) and boiled in 1X Dako pH9 antigen retrieval solution (Agilent, S236784-2) for 10 min. The slides and the hot retrieval solution were left to cool down at room temperature for another 20 min before the slides were rinsed twice in ddH2O. A hydrophobic barrier was drawn around the tissue using the ImmEdge Hydrophobic Barrier pen (Vector Labs, 310018). For slides that were treated with Protease, the tissue was treated with Protease III (Biotechne, 322337) diluted 1:10 with cold PBS and incubated at 40°C in an ACD HybEZ Hybridization System oven (Biotechne, 310013) for 20 min, then rinsed twice with ddH2O. Slides not treated with protease remained in ddH2O throughout this process. Next, endogenous peroxidase was inactivated using 3% H2O2 in PBS and rinsed twice in ddH2O.

Slides were incubated overnight at 40°C with RNAScope probes that detect SIVmac239 vif-env-nef-tar vRNA (Biotechne, 416131-C2) and SIVmac239 gag-pol vDNA (Biotechne, 416141). The next day, slides were washed twice with 0.5X Wash buffer (Biotechne, 310091) for 2 min each. Branched-chain amplification was performed using the Multiplex Fluorescent V2 kit (Biotechne, 323110) with the following conditions, with a 2 x 2 min wash between each step:

1 Amplifier 1, 30 min at 40°C
2 Amplifier 2, 15 min at 40°C
3 Amplifier 3, 30 min at 40°C
4 Channel 1 specific:
  a Amplifier 4, 15 min at 40°C
  b Custom Amplifier 5, 30 min at room temperature
  c Custom Amplifier 5, 15 min at room temperature
  d Biotium Tyramide CF640R deposition, 15 min at room temperature
5 Hydrogen peroxidase block (Biotechne, 323107), 15 min at 40°C
6 Channel 2 specific:
  a Amplifier 4, 15 min at 40°C
  b Custom Amplifier 5, 30 min at room temperature
  c Custom Amplifier 6, 15 min at room temperature
  d Biotium Tyramide CF568 deposition, 15 min at room temperature

All TSA hapten reagents were diluted in an in-house TSA diluent (0.1M Borate, pH 8.5, with 2% w/v Dextran Sulfate Sodium salt and 0.003% H2O2, with the H2O2 added just before the dilution of the tyramide reagent) for 3 minutes at room temperature. We observed that in slides without protease treatment, a higher concentration of CF568 was needed to fully amplify vRNA signals (as determined from FDC-bound SIV vRNA) compared to protease-treated slides. It is important to note that we did not observe any other differences, such as off-target signals and tissue morphological changes, from this increased concentration of CF568. Slides were then rinsed once with 1 x TBS-T, counterstained with DAPI and cover-slipped with #1.5 GOLD SEAL cover glass (EMS, 63791-10) using Prolong Gold Mounting medium (ThermoFisher, P36930). Whole-slide high-resolution fluorescent scans were performed using a Plan-Apochromat 20X objective (NA 0.80) in the Zeiss AxioScan Z.1 slide scanner. DAPI, AF568 and Cy5 (For CF640R) channels were used to acquire images. The exposure time for image acquisition was between 4 and 300 ms.

#### PANINI staining

FFPE tissue paraffin blocks were sectioned onto vectabond treated gold slides at 4 μm thickness on a microtome. Slides were baked for 1h at 70°C and soaked in xylene for 3 x 10min. Standard deparaffinization was performed thereafter (3 x xylene, 3 x 100% EtOH, 2 x 95% EtOH, 1 x 80% EtOH, 1 X 70% EtOH, 3 x H2O) on a linear stainer (Leica Biosystems, ST4020). Epitope retrieval was performed at 97C for 10 min with the Dako Target Retrieval Solution pH 9 (Agilent, S236784-2) on a Lab Vision PT Module (Thermo Fisher Scientific).

Slides were cooled down to 65°C in the PT Module, and left to further cool to room temperature. The region containing the tissue sections were traced out using an ImmEdge PAP pen (Vector Labs, H-4000) before rinsing 2 × 2 min in ddH2O. Tissue sections were then subject to a hydrogen peroxidase block (Biotechne, 322330) at 40°C for 15 min, before 2 x 2 min ddH2O wash. Avidin and Biotin blocks (Biolegend, 927301) were then performed for 15 min each at room temperature, with 2 x 2 min ddH2O washes after each block.

RNAscope probes (see key resources table) were then added for overnight hybridization (∼18 hrs), and all washes from hence forth were performed using RNAscope wash buffer (Biotechne, 310091) for 2 x 2 min at room temperature. Branched-chain amplification using a customized version of the Multiplex Fluorescent Detection Kit v2 (Biotechne, 323110), in which additional amplification was enabled (Amplifiers 5 & 6) for each channel. All amplification reactions were performed at 40°C, except for the following which occur at room temperature: 1) Amplifiers 5 & 6 and 2) Hapten-deposition via tyramine signal amplification (TSA). Reagents for TSA hapten deposition were Biotin (Akoya, NEL749A001KT) and DIG (Akoya, NEL748001KT). All 40°C steps were performed in an ACD HybEZ Hybridization System oven (Biotechne, 310013).

Branched-chain amplification was performed using the Multiplex Fluorescent V2 kit (Biotechne, 323110) with the following conditions, with a 2 x 2 min wash between each step:

1 Amplifier 1, 30 min at 40°C
2 Amplifier 2, 15 min at 40°C
3 Amplifier 3, 30 min at 40°C
4 Channel 1 specific:
  a Amplifier 4, 15 min at 40°C
  b Custom Amplifier 5, 30 min at room temperature
  c Custom Amplifier 5, 15 min at room temperature
  d TSA Biotin (Akoya, NEL749A001KT) deposition, 15 min at room temperature
5 Hydrogen peroxidase block, 15 min at 40°C
6 Channel 2 specific:
  a Amplifier 4, 15 min at 40°C
  b Custom Amplifier 5, 30 min at room temperature
  c Custom Amplifier 6, 15 min at room temperature
  d TSA DIG (Akoya, NEL748001KT) deposition, 15 min at room temperature

The slides were washed for 2 x 5 min with MIBI Wash Buffer (1X TBS-T, 0.1% BSA), then subsequently blocked in Antibody Blocking Buffer (1X TBS-T, 2% Donkey Serum, 0.1% Triton X-100, 0.05% Sodium Azide) for 1 hour before the addition of the antibody cocktail (antibodies diluted in 1X TBS-T, 3% Donkey Serum, 0.05% Sodium Azide) overnight at 4°C. The following day, slides are washed for 3 x 10 min with MIBI Wash Buffer. For MIBI imaging, a 15 min cross-linking is performed with MIBI Crosslinking Buffer (1X PBS containing 4% PFA and 2% glutaraldehyde). Slides are then quenched briefly in 1X TBS-T, before being subjected to a series of washes and dehydration steps (3 x 100mM Tris pH 7.5, 3 x ddH2O, 1 x 70% EtOH, 1 x 80% EtOH, 2 X 95% EtOH, 3 x 100% EtOH). Vectra Polaris and CODEX sample preparation were performed as previous described (Black et al., 2021; Patel et al., 2019; Phillips et al., 2021).

For IF and MIBI cross validation PANINI experiments, sequential glass and gold slides containing both a 3D8 and 174xCEM pellet were processed exactly as described above, with the exception that the 2^nd^ hapten deposited was TSA PLUS Cy3 (Akoya, NEL744001KT). The glass slides also did not undergo a cross-linking step (which is a MIBI-specific processing step), but instead was subject to an anti-mouse secondary antibody 647 (Biolegend, Poly4053) for 1 hour before 3 x 10 min wash MIBI Wash Buffer, DAPI staining, cover-slipped and image processing on a Keyence BZ-X800 microscope with a Nikon CFI Plan Apo λ 20x object (NA 0.75). In all PANINI experiments on gold slides containing SIV-positive and SIV-negative rhesus macaque lymph node tissue sections, glass slide controls containing sequential tissue sections and the 3D8/174xCEM cell pellets were ran in parallel.

#### Vectra polaris data acquisition and processing

Image acquisition was performed using the Vectra Polaris imaging platform (Vectra Polaris, Akoya Biosciences). Representative regions highlighted in this paper were chosen from an initial whole slide scan, and images were acquired at 40x resolution using FITC, Cy3, Texas Red, Cy5 and Opal 780 filters. Images were spectrally separated with a synthetic algorithm in inForm version 2.4.8 (Akoya Biosciences).

### Quantification and statistical analysis

#### CODEX data acquisition and processing

Image acquisition was performed on a four-channel microscope (BZ-X710, Keyence). All raw microscope images were processed with the CODEX Uploader https://github.com/nolanlab/CODEX) for stitching, deconvolution and background subtraction as described (Black et al., 2021).

#### MIBI-TOF data acquisition and processing

Mass images were acquired on a custom alpha-iteration MIBI-TOF mass spectrometer equipped with a duoplasmatron ion source (Ionpath) running research grade oxygen (Airgas, OX R80). All 196 multiplexed images in this study, an accumulation of 19404 individual channel TIFFs, were acquired using the following parameters:

• Pixel dwell time: 12 ms
• Image size: 400 μm x 400 μm at 512 x 512 pixels
• Probe size: ∼400 nm
• Primary ion current: 3.5 nA as measured via a Faraday cup on the sample holder
• Number of depths: 3

MIBI images were extracted and denoised using MIBIAnalysis tools (https://github.com/lkeren/MIBIAnalysis) as previously described (Keren et al., 2018). All three depths were aligned and summed for all downstream analysis. A detailed description of this algorithm can be found here (Baranski et al., 2021).

#### Image segmentation

Cell segmentation was performed using a local implementation of Mesmer, which utilizes the DeepCell library (deepcell-tf 0.6.0) as described (Greenwald et al., 2021; Van Valen et al., 2016). We adapted the included *multiplex_segmentation.py* python script from the deepcell-tf library and imported the neural network weights for prediction from https://deepcell-data.s3-us-west-1.amazonaws.com/model-weights/Multiplex_Segmentation_20200908_2_head.h5). The input for the segmentation were denoised MIBI images for dsDNA (for nuclear features) and CD45 (for membrane features). Signals from these images were capped at the 99.7th percentile. Utilization of model_mpp = 1.8 in the *multiplex_segmentation.py* script uniformly generated the most ideal segmentation results for all the FOVs in this study.

#### MIBI image analysis and cell type annotation

Features from single cells in segmented MIBI images were extracted based on the segmentation generated above and written out as FCS files. FCS files are then uploaded onto CellEngine (Primity Bio) to visually assess data quality and for concatenation after passing the visual check for the presence of dsDNA and Histone H3 nuclear markers. All subsequent analysis is done using R. While all samples were processed experimentally and computationally in parallel, we further ensured normalization of per FOV signal variation by normalization the markers for each cell on a per-FOV basis using the FOV-specific median Histone H3 ion counts. The data was then arcsinh transformed with a cofactor of 1, followed by a capping of the signal 99.9^th^ percentile. Finally, single-cell data was rescaled to a 0 - 1 range for each marker.

Unsupervised classification of cell types was then performed on this scaled data with FlowSOM (Gassen et al., 2015) and cell types were identified from each cluster with marker enrichment modeling (Diggins et al., 2017). The following markers were used for cell type identification: CD16, DC-SIGN, CD4, CD56, CD21, Pax-5, CD163, CD68, CD3, CD20, CD169, CD8a, CD11b, CD36, CD45, MPO, SMA, HLA-DR and CD138. Identified clusters were plotted in 2D space and carefully visually compared with the MIBI multiplexed images to confirm accuracy and specificity of the annotations. Given the downregulation of CD4 by SIV infection, we used CD3^+^ CD8^+^ to determine CD8 T cells and CD3^+^ CD8^-^ CD4^+/-^ to determine CD4 T cells. Clusters that did not meet the accuracy and specificity visual threshold were subject to further iterative clustering.

#### Cellular neighborhood analysis

Cellular Neighborhoods were computationally defined by anchoring each cell and grouping it with its 19 nearest neighbors. The cell type frequencies are counted for each group, across all tissues, and all groups (one group for each single-cell present in this study) and their respective cell type frequencies subject to a k-means clustering of k = 11. Further details on this methodology can be found as previously described (Schürch et al., 2020). The scripts for performing CN identification can be found at: https://github.com/nolanlab/NeighborhoodCoordination.

#### Linear discriminant analysis

Each marker features, with the exception of vDNA and vRNA, were summed for the 20 nearest neighbors (including self) of each cell. These means of these summed marker features were calculated for each animal and CN within these animals. This resulted in 11 CNs from each of the 6 animals, for a total of 66 rows of data. This data was then subject to standardization to a mean of 0 and a variance of 1. Linear Discriminant Analysis was subsequently performed using the *lda* function in the *MASS* R package, with the grouping set to the identifier of each individual animal.

#### Marker correlation analysis

The Pearson or Spearman correlations of marker expressions on cell types were calculated using the *rcorr* function of the Hmisc R package. The Euclidean distance between correlation coefficient values between markers were computed and hierarchically clustered using the *hclust* function of the stats R package.

#### Cell interaction analysis

The Delaunay triangulation of cells were identified by their cartesian XY position within each field of view using default setting from the deldir R package. Interacting cells and their coordinates were extracted from the delsgs output of *deldir*, and the distances between cells joined together by the edge of a Delaunay triangle were calculated within the two-dimensional space according to the following formula:

$$Distance=\;\sqrt{{\left(x_{2}-x_{1}\right)}^{2}+{\left(y_{2}-y_{1}\right)}^{2}}$$

Cell to cell interactions within 100um from one another were identified, resulting in 1390517 interactions of the total 1392033 interactions observed between cells.

To establish a baseline distribution of distances, the same triangulation calculation was performed 1000 times, where for each iteration, the cell and neighborhood identified in each field of view were randomly assigned to existing XY positions. The average distance of a cell-cell interaction in each field of view for each permutation was calculated and this set of expected baseline distances was compared to the observed distances with a Wilcoxon Test.

The fold enrichment of distances between the observed data over the mean distances from the permutation test were calculated as follows:

$$Log_{2}\;fold\;enrichment={\log\;}_{2}\left(\frac{Observed\;mean}{Expected\;mean}\right)$$

The log fold of the distances for each cell type and neighborhood interaction where p-values less than 0.05 were plotted for each group using ggplot2 in R.

#### SIV-infected cells

All cells with a positive vDNA signal were marked as SIV-infected, before visually inspected to ascertain viral signal positivity and cell type annotation accuracy. Predominantly, SIV-infected cells were CD4 T cells, macrophages or Tregs. Rare cases of other cell types (such as B cells or endothelial cells) were deemed to be off-target effects and discarded from further investigation. These infected cells were then further divided into transcriptionally silent (vRNA = 0) or active (vRNA > 0) for the purpose of this study. Tregs were removed from all further analysis due to their small representation (n = 94) compared to the other 2 groups (CD4 T cells, n = 543; macrophages, n = 277).

#### Random forest classification

A random forest classifier was used to examine if features of the tissue microenvironment could be used to identify transcriptionally silent and active SIV cells. Optimal parameters for the random forest model were identified using *trainControl* from the caret R package. We randomly sampled 63.2% of the data as the training/tuning group and applying the classifier to the remaining 36.8% as the validation data to predict a cell’s reactivation status. The performance is reported as the median value from 100 repetitions and was evaluated by calculating the true positive rates, false positive rates, and the AUC of the resulting ROC as previously described (Robin et al., 2011). The predicted probabilities were then compared to the true reactivation status using a Wilcoxon test. Note that both vRNA and NF-κB-p100 markers were removed from features used for the random forest classifier as they were molecular determinants of viral transcription.

#### Binned anchor plot analysis

A schematic of the anchor plot analysis is depicted in Figure 7C. All cells within a 100um range were extracted (1 pixel = 0.78125um), and the frequency of cell type and marker expression summed in 10um increments. These values were then divided by the number of cells, to normalize for differences in cell numbers in a radial spread from the center “anchor” cell. The 95% confidence interval for each binned value was then calculated and plotted along with the mean. Anchor plots were segregated by 1. Cell type (infected CD4 T cell or infected macrophage) and 2. Transcription status (transcriptionally silent or active).

#### Data visualization

All pseudo-colored MIBI images were visualized using a Nolan lab specific instance of the MIBITracker (Ionpath). Figures 1A, 4A, 6E, 7C, and 7G were generated in part using Biorender. All other plots in this manuscript were generated with the *ggplots2* R package (Wickham, 2016).

## Data Availability

• All processed MIBI multiplexed imaging data produced in this paper are available via the MIBItracker Public Accession Portal at the following link: https://mibi-share.ionpath.com/. • All original code has been deposited at Zendo and is publicly available as of the date of publication. DOIs are listed in the key resources table. • Any additional information required to reanalyze the data reported in this paper is available from the lead contact upon request.

## Supplemental information

Document S1. Figures S1–S7

Table S1. Animal information, related to Figures 1, 2, 3, 4, 5, 6, and 7

Table S2. MIBI antibody information, related to Figures 1, 2, 3, 4, 5, 6, and 7

Document S2. Article plus supplemental information
