# Supplementary material for: Combined protein and nucleic acid imaging reveals virus-dependent B cell and macrophage immunosuppression of tissue microenvironments
Source: Immunity. 2022 Jun 14;55(6):1118–1134.e8. doi: 10.1016/j.immuni.2022.03.020 (PMC9220319; doi:10.1016/j.immuni.2022.03.020)
Supplement: Document S1. Figures S1–S7 [file mmc1.pdf]

Immunity, Volume *55*

Supplemental information

Combined protein and nucleic acid imaging reveals virus-dependent B cell and macrophage immunosuppression of tissue microenvironments

Sizun Jiang, Chi Ngai Chan, Xavier Rovira-Clavé, Han Chen, Yunhao Bai, Bokai Zhu, Erin McCaffrey, Noah F. Greenwald, Candace Liu, Graham L. Barlow, Jason L. Weirather, John Paul Oliveria, Tsuguhisa Nakayama, Ivan T. Lee, Matthias S. Matter, Anne E. Carlisle, Darci Philips, Gustavo Vazquez, Nilanjan Mukherjee, Kathleen Busman-Sahay, Michael Nekorchuk, Margaret Terry, Skyler Younger, Marc Bosse, Janos Demeter, Scott J. Rodig, Alexandar Tzankov, Yury Goltsev, David Robert McIlwain, Michael Angelo, Jacob D. Estes, and Garry P. Nolan

Figure S1

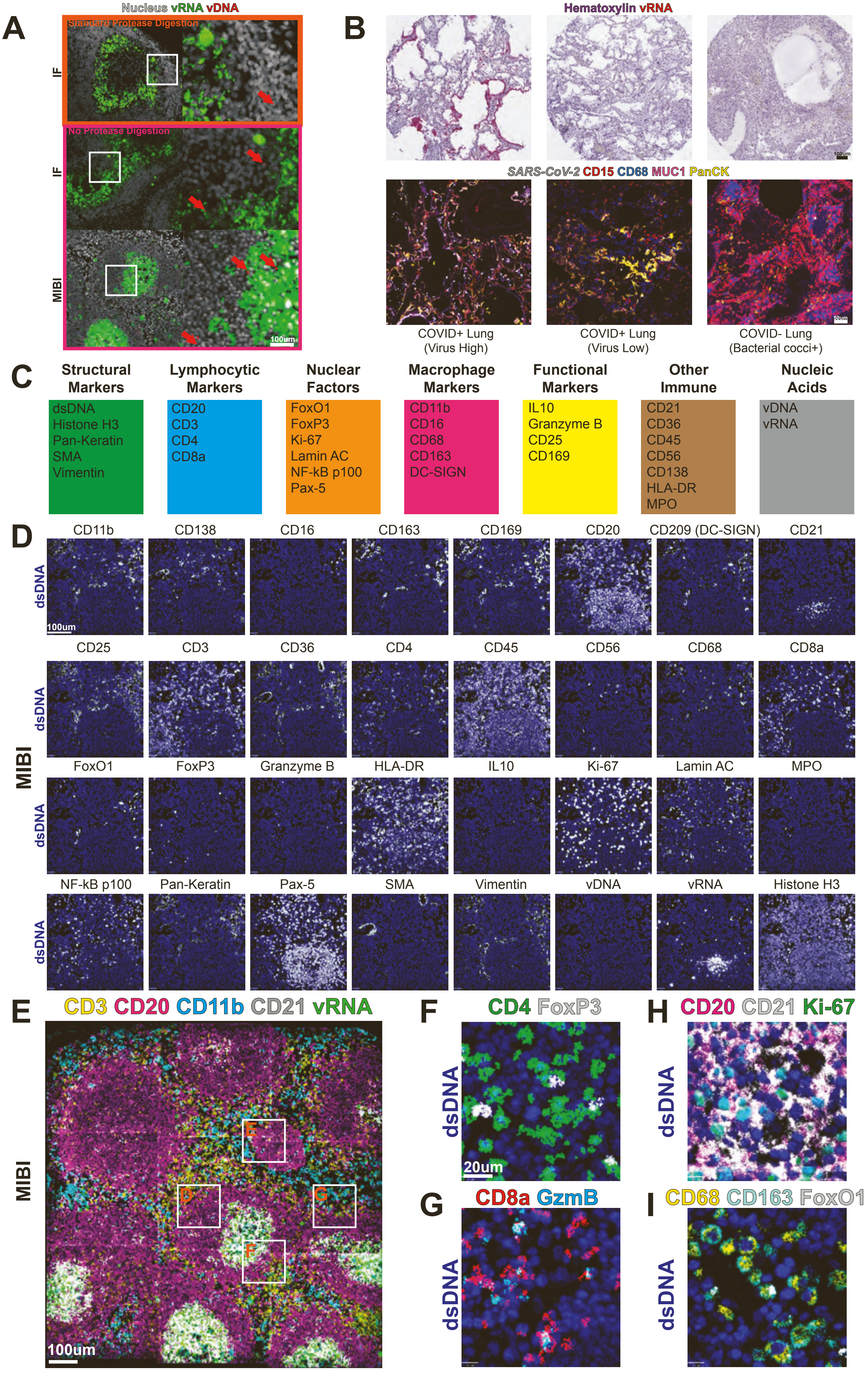
A
Nucleus vRNA vDNA
Standard Protease Digestion
IF
No Protease Digestion
IF
MIBI
100um
B
Hematoxylin vRNA
100um
SARS-CoV-2 CD15 CD68 MUC1 PanCK
50um
COVID+ Lung
(Virus High)
COVID+ Lung
(Virus Low)
COVID- Lung
(Bacterial cocci+)
C
Structural Markers
dsDNA
Histone H3
Pan-Keratin
SMA
Vimentin
Lymphocytic Markers
CD20
CD3
CD4
CD8a
Nuclear Factors
FoxO1
FoxP3
Ki-67
Lamin AC
NF-kB p100
Pax-5
Macrophage Markers
CD11b
CD16
CD68
CD163
DC-SIGN
Functional Markers
IL10
Granzyme B
CD25
CD169
Other Immune
CD21
CD36
CD45
CD56
CD138
HLA-DR
MPO
Nucleic Acids
vDNA
vRNA
D
MIBI
dsDNA
CD11b
CD138
CD16
CD163
CD169
CD20
CD209 (DC-SIGN)
CD21
100um
CD25
CD3
CD36
CD4
CD45
CD56
CD68
CD8a
FoxO1
FoxP3
Granzyme B
HLA-DR
IL10
Ki-67
Lamin AC
MPO
NF-kB p100
Pan-Keratin
Pax-5
SMA
Vimentin
vDNA
vRNA
Histone H3
E
CD3 CD20 CD11b CD21 vRNA
MIBI
E
D
G
F
100um
F
CD4 FoxP3
dsDNA
20um
G
CD8a GzmB
dsDNA
H
CD20 CD21 Ki-67
dsDNA
I
CD68 CD163 FoxO1
dsDNA

Figure S2

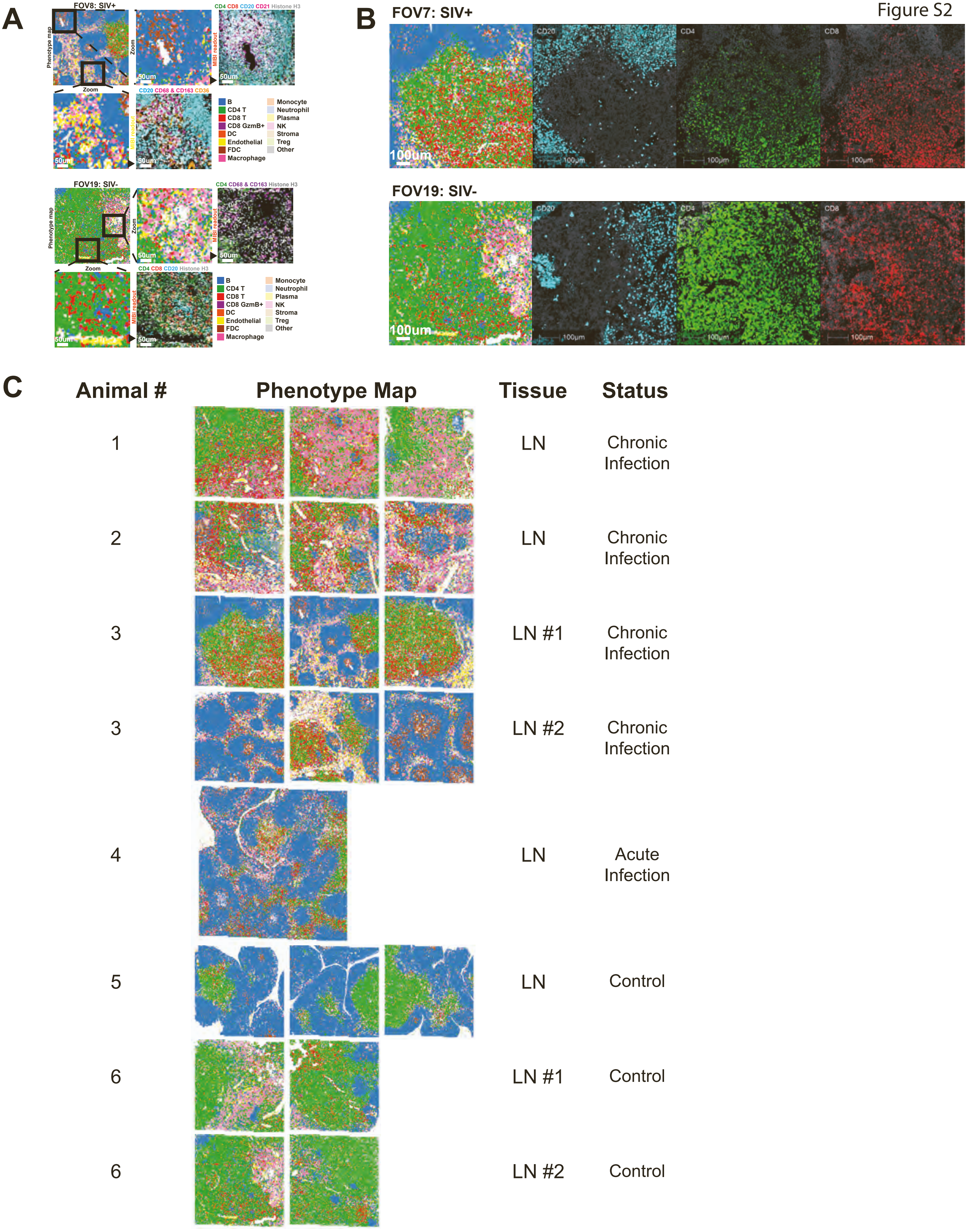
A
FOV8: SIV+
Phenotype map
Zoom
MIBI readout
CD4 CD8 CD20 CD21 Histone H3
CD20 CD68 & CD163 CD36
50um
B
CD4 T
CD8 T
CD8 GzmB+
DC
Endothelial
FDC
Macrophage
Monocyte
Neutrophil
Plasma
NK
Stroma
Treg
Other
FOV19: SIV-
CD4 CD68 & CD163 Histone H3
CD4 CD8 CD20 Histone H3
B
FOV7: SIV+
CD20
CD4
CD8
100um
FOV19: SIV-
C
Animal #
Phenotype Map
Tissue
Status
1
LN
Chronic Infection
2
LN
Chronic Infection
3
LN #1
Chronic Infection
3
LN #2
Chronic Infection
4
LN
Acute Infection
5
LN
Control
6
LN #1
Control
6
LN #2
Control

Figure S3

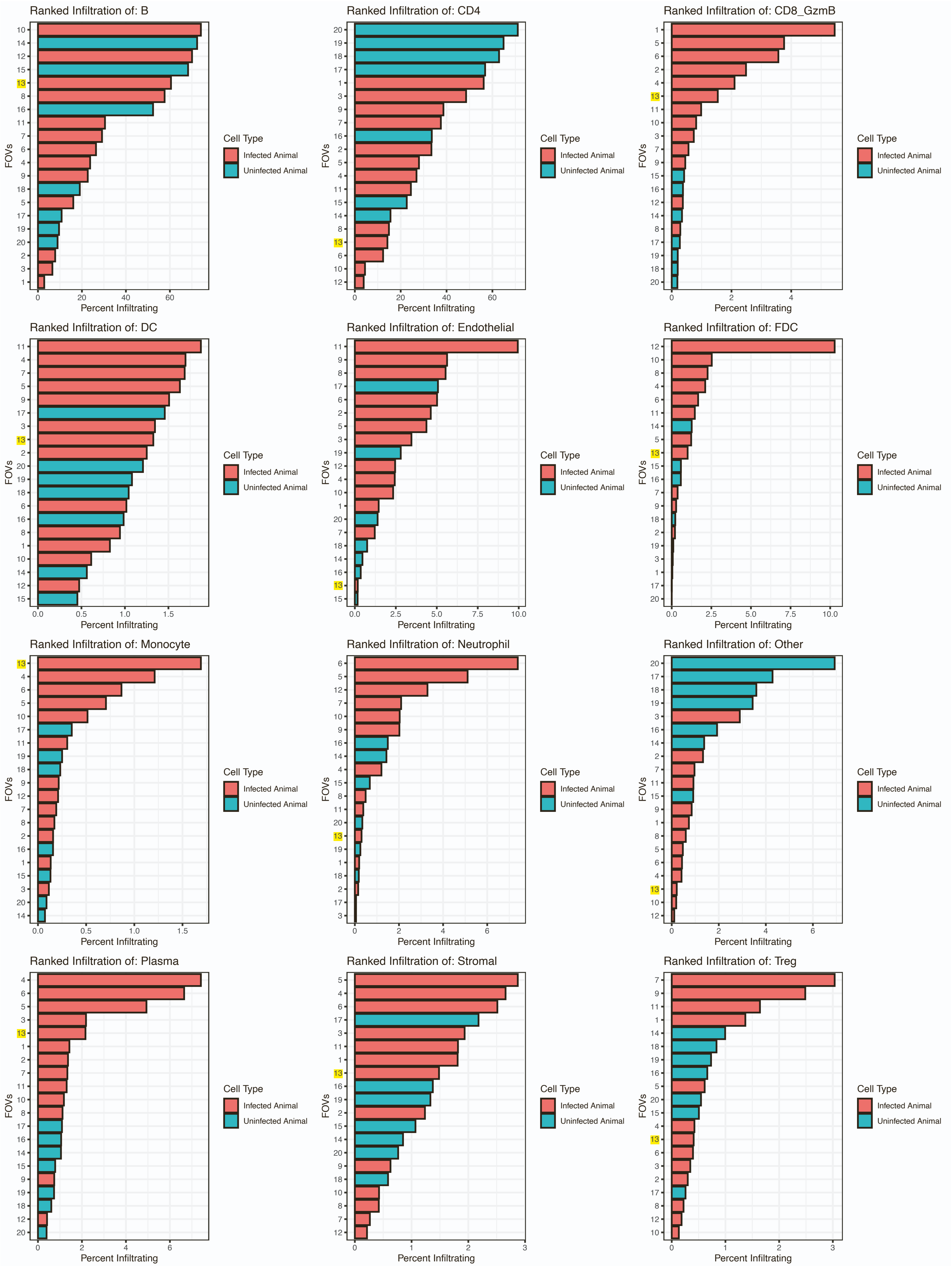
Ranked Infiltration of: B
Ranked Infiltration of: CD4
Ranked Infiltration of: CD8_GzmB
Ranked Infiltration of: DC
Ranked Infiltration of: Endothelial
Ranked Infiltration of: FDC
Ranked Infiltration of: Monocyte
Ranked Infiltration of: Neutrophil
Ranked Infiltration of: Other
Ranked Infiltration of: Plasma
Ranked Infiltration of: Stromal
Ranked Infiltration of: Treg
FOVs
Percent Infiltrating
Cell Type
Infected Animal
Uninfected Animal

Figure S4

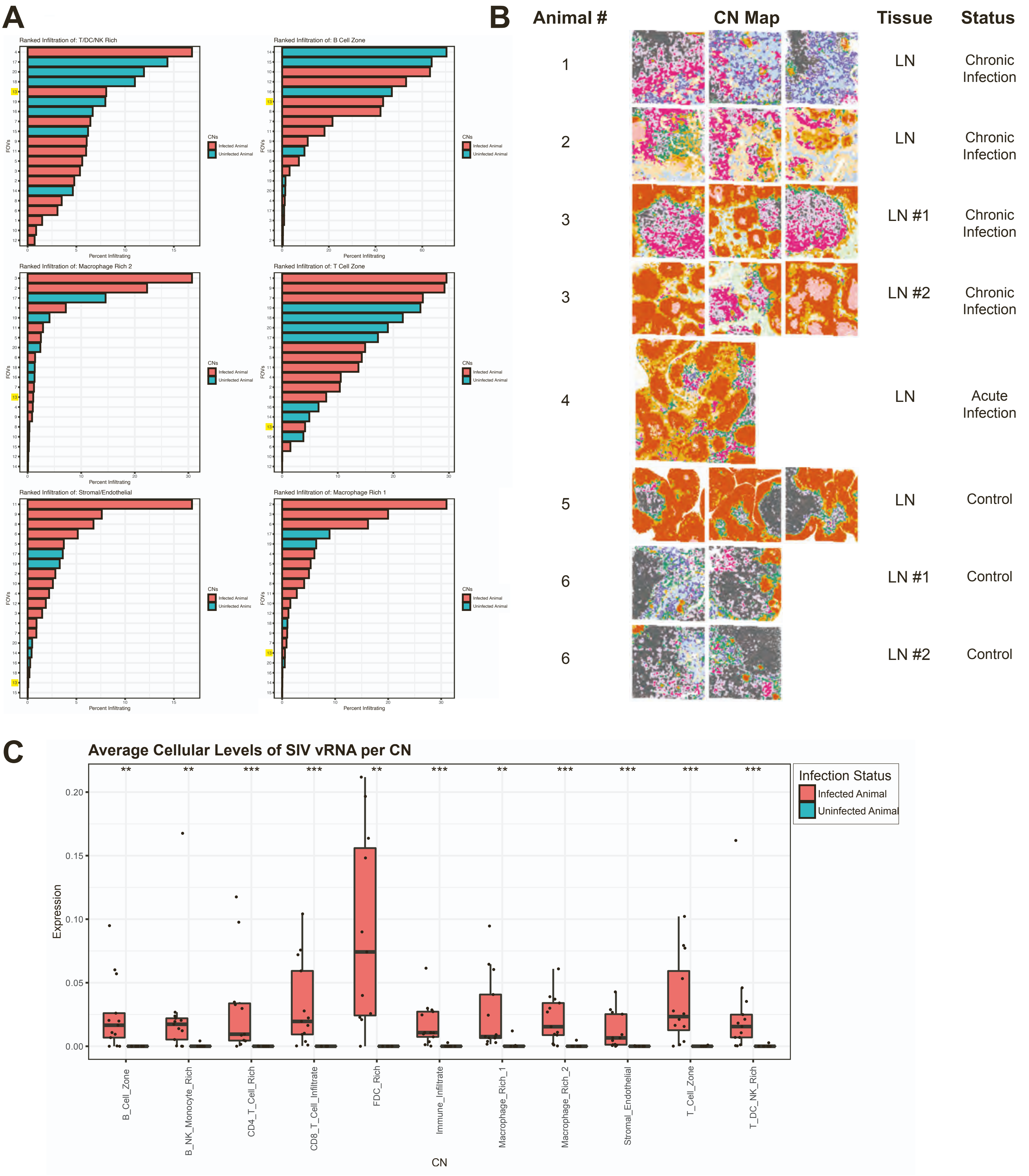
A
Ranked Infiltration of: T/DC/NK Rich
Ranked Infiltration of: B Cell Zone
Ranked Infiltration of: Macrophage Rich 2
Ranked Infiltration of: T Cell Zone
Ranked Infiltration of: Stromal/Endothelial
Ranked Infiltration of: Macrophage Rich 1
FOVs
Percent Infiltrating
CNs
Infected Animal
Uninfected Animal
B
Animal #
CN Map
Tissue
Status
1
LN
Chronic Infection
2
LN
Chronic Infection
3
LN #1
Chronic Infection
3
LN #2
Chronic Infection
4
LN
Acute Infection
5
LN
Control
6
LN #1
Control
6
LN #2
Control
C
Average Cellular Levels of SIV vRNA per CN
Expression
CN
Infection Status
Infected Animal
Uninfected Animal
B_Cell_Zone
B_NK_Monocyte_Rich
CD4_T_Cell_Rich
CD8_T_Cell_Infiltrate
FDC_Rich
Immune_Infiltrate
Macrophage_Rich_1
Macrophage_Rich_2
Stromal_Endothelial
T_Cell_Zone
T_DC_NK_Rich

Figure S5

A

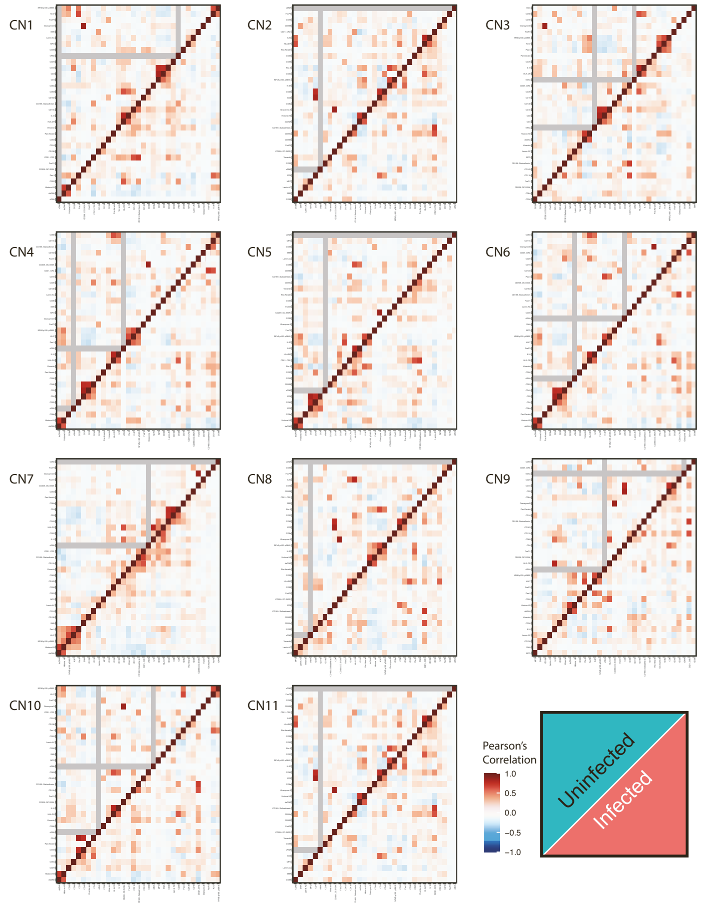
CN1
CN2
CN3
CN4
CN5
CN6
CN7
CN8
CN9
CN10
CN11
Pearson's Correlation
1.0
0.5
0.0
−0.5
−1.0
Uninfected
Infected

B

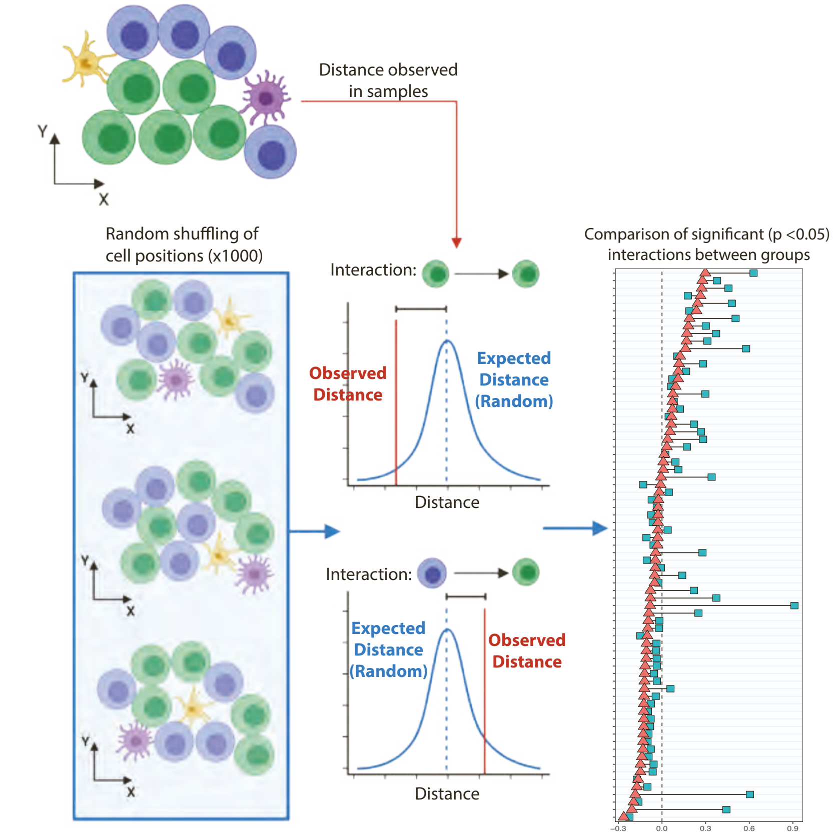
Distance observed in samples
Y
X
Random shuffling of cell positions (x1000)
Interaction:
Observed Distance
Expected Distance (Random)
Distance
Interaction:
Expected Distance (Random)
Observed Distance
Distance
Comparison of significant (p <0.05) interactions between groups
−0.3
0.0
0.3
0.6
0.9

Figure S6

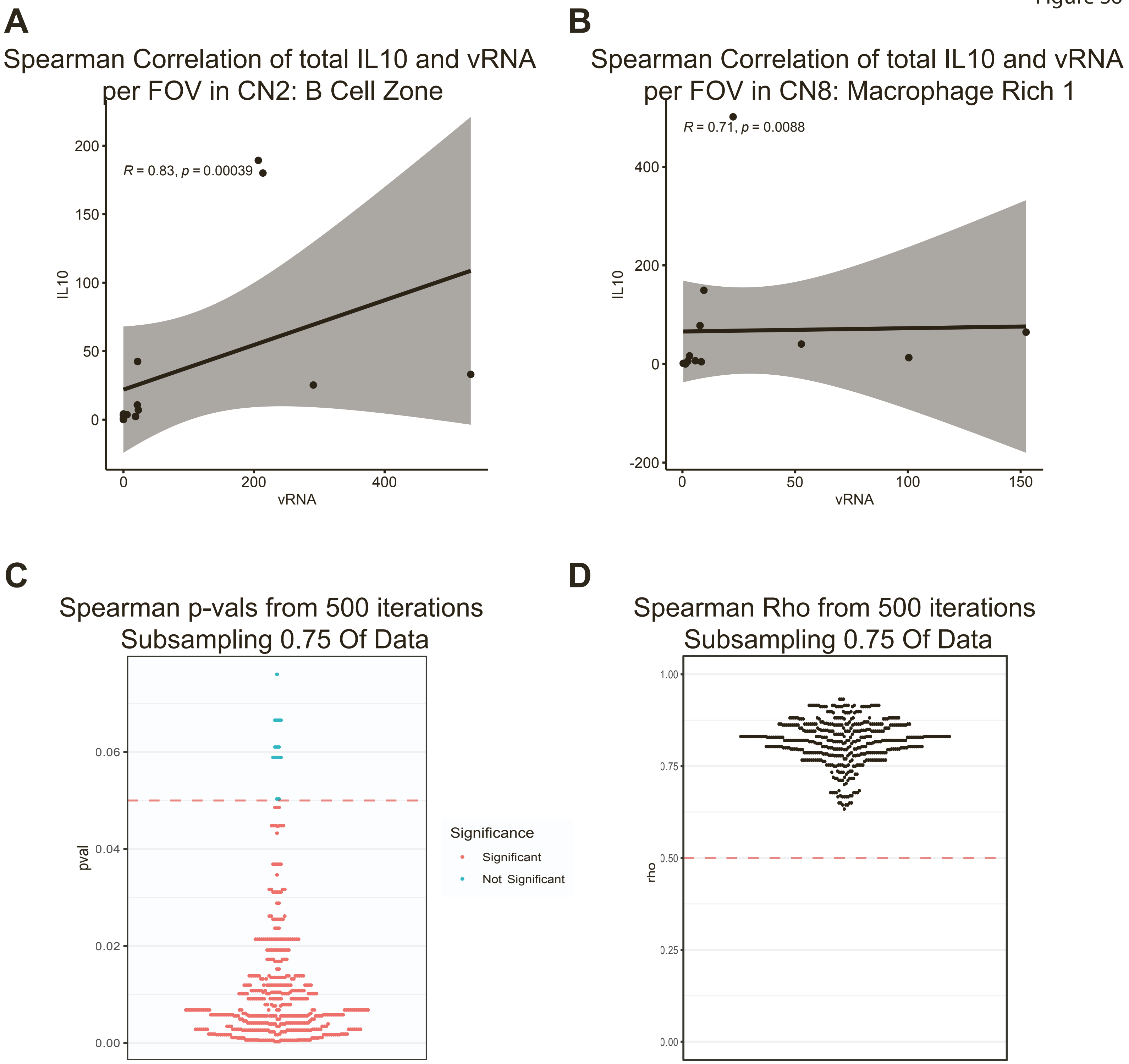
A
Spearman Correlation of total IL10 and vRNA per FOV in CN2: B Cell Zone
R = 0.83, p = 0.00039
IL10
vRNA
B
Spearman Correlation of total IL10 and vRNA per FOV in CN8: Macrophage Rich 1
R = 0.71, p = 0.0088
IL10
vRNA
C
Spearman p-vals from 500 iterations Subsampling 0.75 Of Data
pval
Significance
Significant
Not Significant
D
Spearman Rho from 500 iterations Subsampling 0.75 Of Data
rho

Figure S7

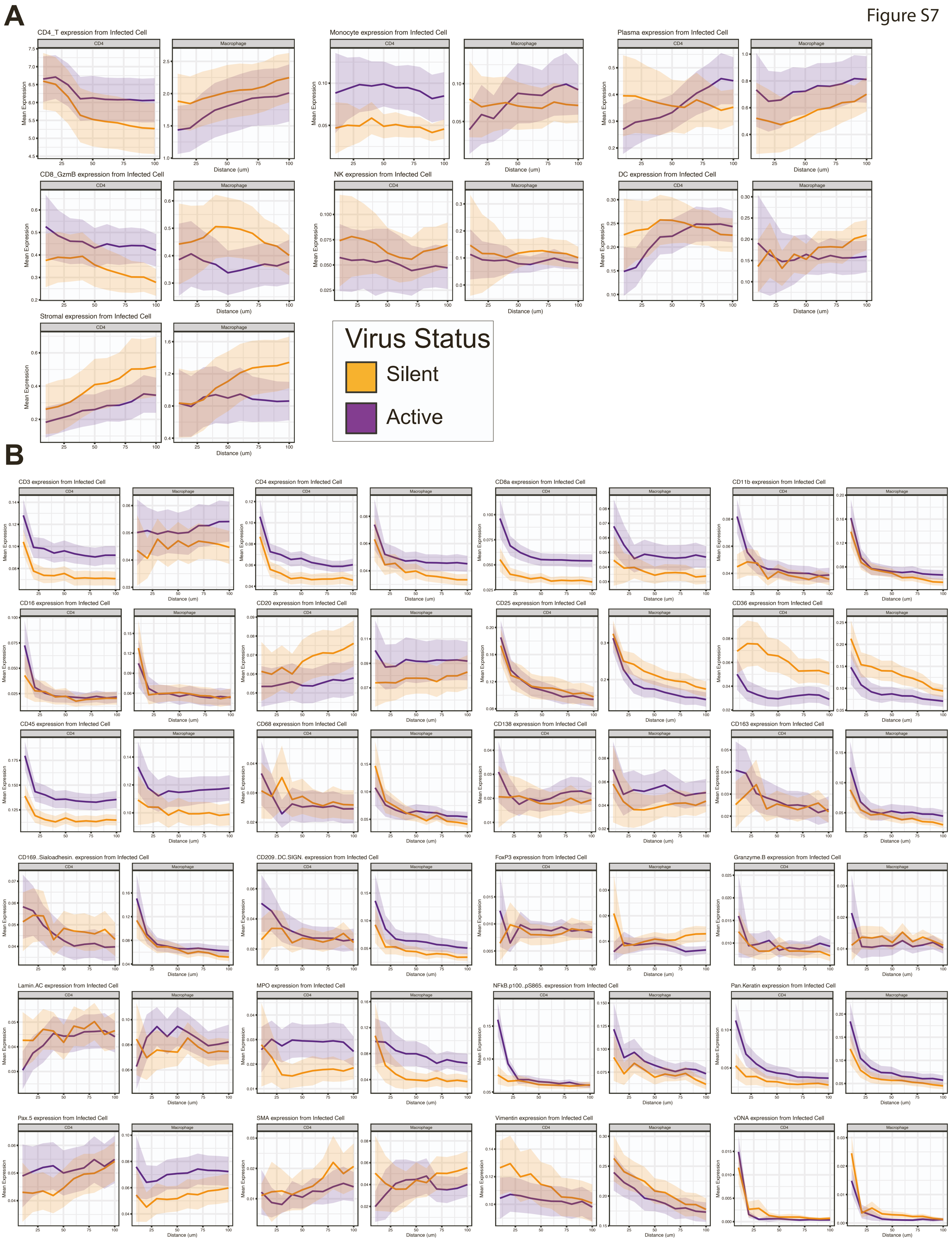
A
CD4_T expression from Infected Cell
Monocyte expression from Infected Cell
Plasma expression from Infected Cell
CD8_GzmB expression from Infected Cell
NK expression from Infected Cell
DC expression from Infected Cell
Stromal expression from Infected Cell
CD4
Macrophage
Mean Expression
Distance (um)
Virus Status
Silent
Active
B
CD3 expression from Infected Cell
CD4 expression from Infected Cell
CD8a expression from Infected Cell
CD11b expression from Infected Cell
CD16 expression from Infected Cell
CD20 expression from Infected Cell
CD25 expression from Infected Cell
CD36 expression from Infected Cell
CD45 expression from Infected Cell
CD68 expression from Infected Cell
CD138 expression from Infected Cell
CD163 expression from Infected Cell
CD169..Sialoadhesin. expression from Infected Cell
CD209..DC.SIGN. expression from Infected Cell
FoxP3 expression from Infected Cell
Granzyme.B expression from Infected Cell
Lamin.AC expression from Infected Cell
MPO expression from Infected Cell
NFkB.p100..pS865. expression from Infected Cell
Pan.Keratin expression from Infected Cell
Pax.5 expression from Infected Cell
SMA expression from Infected Cell
Vimentin expression from Infected Cell
vDNA expression from Infected Cell

SUPPLEMENTAL FIGURE LEGENDS

Figure S1: Validation of the PANINI Approach and Antibody Staining Specificity. Related to Figure 1

(A) Representative images of SIV-positive lymph nodes subjected to standard protease digestion step (top) after epitope retrieval or no protease treatment (middle and bottom) after epitope retrieval. SIV vDNA (red) and vRNA (green) were detected using ISH followed by hapten deposition, and subsequently imaged using either IF or MIBI. Cells harboring integrated virus are indicated with red arrows.

(B) Top: single-plex brightfield *in situ* hybridization for SARS-CoV-2 Spike mRNA on COVID-19 positive and negative lung tissues, as indicated at the bottom of the columns. Bottom: CODEX on lung samples from the same tissues shown above for the SARS-CoV-2 Spike mRNA (SARS-CoV-2, white), neutrophil marker (CD15, red), macrophage marker (CD68, blue), type II pneumocytic marker (MUC1, magenta) and epithelia marker pan-cytokeratin (PanCK, yellow).

(C) The validated rhesus macaque compatible marker panel used in this study.

(D) Images for each of the 33 markers depicted in pairwise fashion with dsDNA (blue). The field-of-view (FOV) represented here is a germinal center within an SIV-positive lymph node.

(E) A large FOV representing a 1.2 mm x 1.2 mm region of a SIV-positive lymph node with several lineage-specific markers. White boxes indicated regions magnified in the following panels.

(F) A magnified region of panel C containing $CD4^+$ and $FoxP3^+$ T cells.

(G) A magnified region of panel C containing $CD8^+$ and granzyme B^+ T cells.

(H) A magnified region of panel C containing $CD20^+$, $CD21^+$ and Ki-67^+ B cells and FDCs.

(I) A magnified region of panel C containing $CD68^+$, $CD163^+$, and $FoxO1^+$ macrophages.

Figure S2: Validation of the Unsupervised Computational Approach for Cell Type Identification from PANINI-MIBI Multiplexed Imaging Data. Related to Figure 2

(A) Phenotype maps of two FOVs with two magnified regions of both the phenotype map and the paired pseudo-colored MIBI image with lineage-specific markers to validate the computationally determined cell phenotypes.

(B) Representative FOVs from an SIV-positive and a control lymph node with three subjacent tissue sections that were stained for CD20 (blue), CD4 (green), and CD8 (red) and imaged using an IF microscope for orthogonal validation of PANINI-MIBI staining. The antibody clones and staining conditions used for the IF validation were identical to PANINI-MIBI.

(C) Phenotype maps of all 20 FOVs and their associated tissue sources. All FOVs are 1.2 mm x 1.2 mm with the exception of that from Animal 4 (2 mm x 2 mm).

Figure S3: Orchestrated Immune Composition and Responses to SIV Infection. Related to Figure 3

Ranked bar plots showing the percent infiltration of each cell type for the 20 FOVs with bars colored by infection status. The yellow box indicates the acutely infected animal.

Figure S4: Cell Neighborhoods Capture Virus-Infection Induced Changes of Tissue Microenvironments. Related to Figure 4

(A) Ranked bar plots showing the percent infiltration of each CN across the 20 FOVs with bars colored by infection status.
(B) CN maps of all 20 FOVs and their associated tissue sources. All FOVs are 1.2 mm x 1.2 mm with the exception of that from Animal 4 (2 mm x 2 mm). The yellow box indicates the acutely infected animal.
(C) The mean SIV vRNA quantities per CN. Each dot represents an individual FOV from an infected (orange) or uninfected (teal) animal. Non-paired Wilcoxon test: ns, not significant; *, $p<0.05$; **, $p<0.01$; ***, $p<0.001$.

Figure S5: Global Tissue Reorganization during SIV-infection. Related to Figure 5
(A) Heatmaps of pairwise Pearson's correlations of markers across each individual cell within each CN for infected (top left) and healthy (bottom right) animals.
(B) A cartoon representation for how the permutation test for cell-cell or CN-CN interaction is computed.

Figure S6: Statistical Confirmation that IL-10 positivity correlates with viral RNA quantities and Macrophage Polarization. Related to Figure 6
(A and B) Plots of Spearman's correlations between IL-10 and vRNA quantification in (A) CN2 and (B) CN8 after dropping out a postulated outlier.
(C and D) Plots of Spearman's correlation between IL-10 and vRNA quantification pertaining to their (C) p-values and (D) rho coefficients after 500 iterations of subsampling 75% of the data. Two- sample correlation test between Spearman correlations: Significant = $p<0.05$.

Figure S7: Spatial Determinants for Retroviral Transcription State. Related to Figure 7
(A and B) Anchor plots of (A) mean cell type quantifications and (B) mean marker expression around infected $CD4^+$ T cells (top) or macrophages (bottom). Orange indicates transcriptionally silent cells, and purple indicates transcriptionally active cells. The thick colored lines represent the mean values, and the light regions around these lines depict the 95% confidence intervals. The infected cells were anchored at 0 μm, and the plot ends at 100 μm
